# The impact of CSF1R inhibitor-mediated microglial depletion in rodent models of Alzheimer’s and Parkinson’s disease: a systematic review and meta-analysis

**DOI:** 10.3389/fnagi.2026.1733682

**Published:** 2026-02-25

**Authors:** Ana Flavia F. Ferreira, Ana Caroline Santos-Silva, Beatriz Gangale Muratori, Luiz Roberto Britto

**Affiliations:** Department of Physiology and Biophysics, Institute of Biomedical Sciences, University of São Paulo, São Paulo, Brazil

**Keywords:** CSF1-R inhibition, microglial depletion, microglial repopulation, neurodegeneration, neuroprotection

## Abstract

Microglia are central nervous system immune cells that support brain homeostasis but can adopt harmful roles in neurodegenerative diseases such as Alzheimer’s disease (AD) and Parkinson’s disease (PD), depending on the disease stage and progression. Thus, targeting microglia through depletion has emerged as a potential therapeutic approach. This systematic review and meta-analysis aim to evaluate the effects of microglial depletion using colony-stimulating factor 1 receptor (CSF1R) inhibitors, such as PLX3397 and PLX5622, in preclinical models of AD and PD. Twenty-six AD and seventeen PD preclinical studies were selected. In PD models, most studies reported neuroprotective effects after microglial depletion, though a few showed detrimental outcomes, particularly with shorter depletion protocols. Notably, almost all studies induced microglial depletion prior to or during disease onset, underscoring a major research gap. Behavioral results were contradictory, as some reported beneficial effects while others showed no effect or worsened behavior. In AD models, results were more variable, but many studies observed reduced neuroinflammation, improved cognition, and decreased amyloid-beta and tau pathology. Meta-analyses showed no overall reduction in dopaminergic neuron loss in PD or amyloid-beta levels in AD, though longer depletion protocols showed more favorable trends in both diseases. Despite the few reports, repopulation following microglial depletion may constitute a promising approach. Microglial depletion, via PLX3397 and PLX5622, may offer therapeutic potential for both AD and PD, although high heterogeneity and variability among studies are a clear limitation. Further studies are needed, particularly those assessing post-onset intervention, sex-specific effects, and broader behavioral and pathological endpoints to better understand the therapeutic potential of microglial modulation.

**Systematic review registration:**

https://www.crd.york.ac.uk/prospero/, identifier CRD420251075163.

## Graphical abstract

**Figure:**
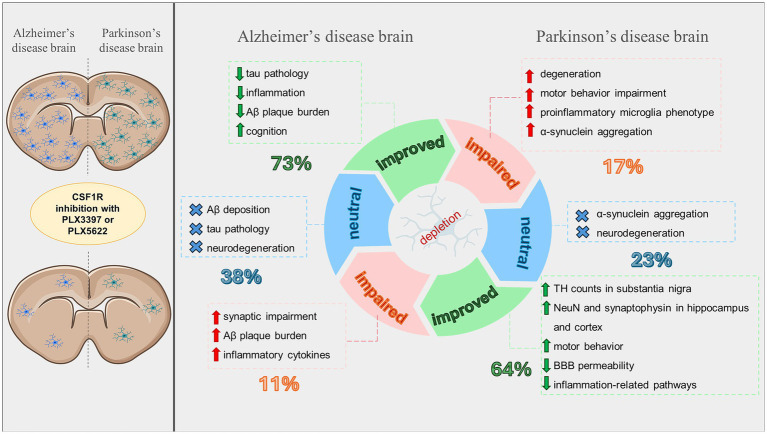

## 1 Introduction

As the global population continues to grow and age, deaths and disability caused by neurological disorders are increasing, making them a major global public health concern (World Health Organization, 2023). Among these disorders, Alzheimer’s (AD) and Parkinson’s disease (PD), in particular, stand out for their high accelerating rates (Li et al., 2025; Xu et al., 2025). AD, the most common cause of dementia, is characterized by two main pathological hallmarks: deposits of misfolded amyloid-beta (Aβ) peptides that, with the disease progression, aggregates into Aβ plaques, and the presence of hyperphosphorylated tau (p-Tau) that forms the intraneuronal neurofibrillary tangles (NTFs) (Möller and Graeber, 1998; Blennow et al., 2006). PD is a progressive movement disorder classically characterized by the triad of motor symptoms: bradykinesia, tremors, and rigidity (Parkinson, 2002). The loss of dopaminergic neurons from the substantia nigra (SN) and the accumulation of the *α*-synuclein protein, which later can aggregate and form the Lewy bodies, are the two main pathological hallmarks of PD (Poewe et al., 2017). In addition, both diseases share a common pathological signal: the inflammatory component.

Inflammation is a physiological response to injury, tissue damage, pathogen invasion, and other insults (Leng and Edison, 2021). In the brain, neuroinflammation is primarily mediated by microglia, the main immune cells of the central nervous system, which regulate synapses, tissue repair, neurogenesis, myelination, immune surveillance, and cytokine production (Yang et al., 2025). Microglia also phagocytose toxins and proteins such as amyloid plaques and *α*-synuclein. In chronic diseases like AD and PD, however, they can shift from protective roles to aberrant, detrimental functions that exacerbate pathology (Heneka et al., 2025; Tansey et al., 2022). Despite extensive research (Hopperton et al., 2018; Qu et al., 2023), the mechanisms driving this phenotypic switch remain unclear, highlighting the therapeutic potential of selectively targeting harmful microglial states. Microglial survival depends on colony-stimulating factor 1 receptor (CSF1R), which regulates their proliferation, migration, differentiation, and survival (Chitu et al., 2016). Pharmacological inhibition of CSF1R was shown to eliminate virtually all microglia in the brain (Elmore et al., 2014), and two particular inhibitors have been often used as a depletion strategy, namely PLX3397 (Pexidartinib) and PLX5622 (Elmore et al., 2014; Spangenberg et al., 2019). Initially investigated as antitumor agents (Wen et al., 2023), PLX3397 is FDA-approved for tenosynovial giant cell tumor as TURALIO® (Lamb, 2019; Food and Drug Administration (FDA), 2019). Recently, these inhibitors have gained attention for their potential to modulate microglia in AD and PD.

Others have previously reviewed CSF1R inhibitors in different contexts (Guenoun et al., 2025; Basilico et al., 2022; Cannarile et al., 2017), but no study has systematically reviewed and meta-analyzed before the effects of microglial modulation by PLX3397 and PLX5622 in AD and PD. In the present study, our aim was to delve into the literature about PLX3397 and PLX5622 in the neurodegenerative disease context, with a focus on AD and PD. This review can help to clarify the dual role of microglia in the two most common neurodegenerative diseases, identifying the microglial depletion protocols that have been used, highlighting the gaps and limitations in the field, contributing to the experimental design of future studies and, ultimately, placing those compounds as potential therapeutic strategies for AD and PD patients.

## 2 Methods

### 2.1 Study guidelines and registration

The Preferred Reporting Items for Systematic reviews and Meta-Analyses (PRISMA) statement was followed as a guideline for this systematic review (Page et al., 2020). Protocol details were registered in the International Prospective Register of Systematic Reviews (PROSPERO) database (https://www.crd.york.ac.uk/prospero/) (Booth et al., 2012; Bannach-Brown et al., 2024) under the reference number CRD420251075163.

### 2.2 Searching and screening

Two researchers (AFFF and ACSS) conducted the search in the following databases: MEDLINE (via PubMed); EMBASE; and Web of Science. Searches were not restricted by date of publication or language. Database search strategies for each database are available in *Supplementary Material 1.* The search was conducted on January 1st 2025, with an updated search on July 13th 2025. The following key words were used: PLX3397 or PLX5622 or CSF1R Inhibitor or Pexidartinib.

### 2.3 Study selection

Studies were selected by two reviewers (AFFF and ACSS), independently. Using the Mendeley Reference Manager (Mendeley Desktop 1.19.5 Installers), duplicates were removed and the title and abstract of the studies were first screened according to the following exclusion criteria: (1) not available in English; (2) does not include *in vivo* rodent study; (3) not an original paper (e.g., review, case report, conference, book chapter); (4) not a neurodegenerative disease; and (5) not PLX3397 or PLX5622 as a treatment. The selected studies were included in a second phase screening, in which full-texts were assessed by the two reviewers. The studies were excluded if: (1) met with the previous exclusion criteria, (2) no full-text available, (3) pre-print studies, (4) no vehicle-treated separated control animals; or (5) only combined treatment. All discrepancies were solved between the two reviewers by open discussions.

### 2.4 Data extraction

Two reviewers (AFFF and ACSS) independently read thoroughly all selected articles and filled tables with the following content: type of disease; animal strain, sex, and age; disease animal model characteristics (mutations/neurotoxin; administration route, dose and frequency); treatment characteristics (PLX type, dose, protocol, duration, and route of administration); microglia removal time; percentage of microglial removal; behavioral tests and outcomes; molecular assays and outcomes. A third reviewer (BGM) independently revised all the collected data.

### 2.5 Quality assessment

The Systematic Review Centre for Laboratory Animal Experimentation (SYRCLE) RoB tool, a specifically developed tool for preclinical animal studies, was used by two first reviewers (AFFF and ACSS) to assess the studies quality (Hooijmans et al., 2014). The SYRCLE’s RoB tool contains 10 entries that accesses the risk of bias different bias of animal intervention studies, such as selection, performance, detection, and reporting biases. The entries were filled with: yes (low risk of bias), no (high risk of bias), or “?” (unclear risk of bias).

### 2.6 Data analysis and synthesis

All selected studies were narratively synthesized and summarized in tables. Studies were included in the meta-analysis if at least three of them provided comparable data. When numerical data were not explicitly reported in the text or tables and were available only in graphical form, means and standard deviations were estimated by measuring graphical values using the digital ruler tool in Adobe Acrobat Reader software, with the same procedure applied consistently across all figures (Bolzan and Lino de Oliveira, 2022). The RevMan Calculator (Cochrane Library, London, United Kingdom) was employed to compute standard deviations when they were not provided. For studies involving multiple experimental groups assessed with different instruments or targeting different brain regions, group means and standard deviations were combined (Borenstein, 2022). Meta-analyses were performed using a random-effects model to account for variability between studies. Effect sizes were expressed as standardized mean differences (SMDs) with corresponding 95% confidence intervals (CIs). Statistical significance was set at *p* ≤ 0.05. Heterogeneity among studies was assessed using the I^2^ statistics. Subgroup analyses were conducted to explore differences in effect sizes based on intervention duration (Higgins et al., 2024; Singulani et al., 2024). All statistical analyses were carried out using Review Manager (RevMan), version 5.4.1 (Cochrane Collaboration, London, United Kingdom).

## 3 Results

### 3.1 Studies selection

**Figure 1:**
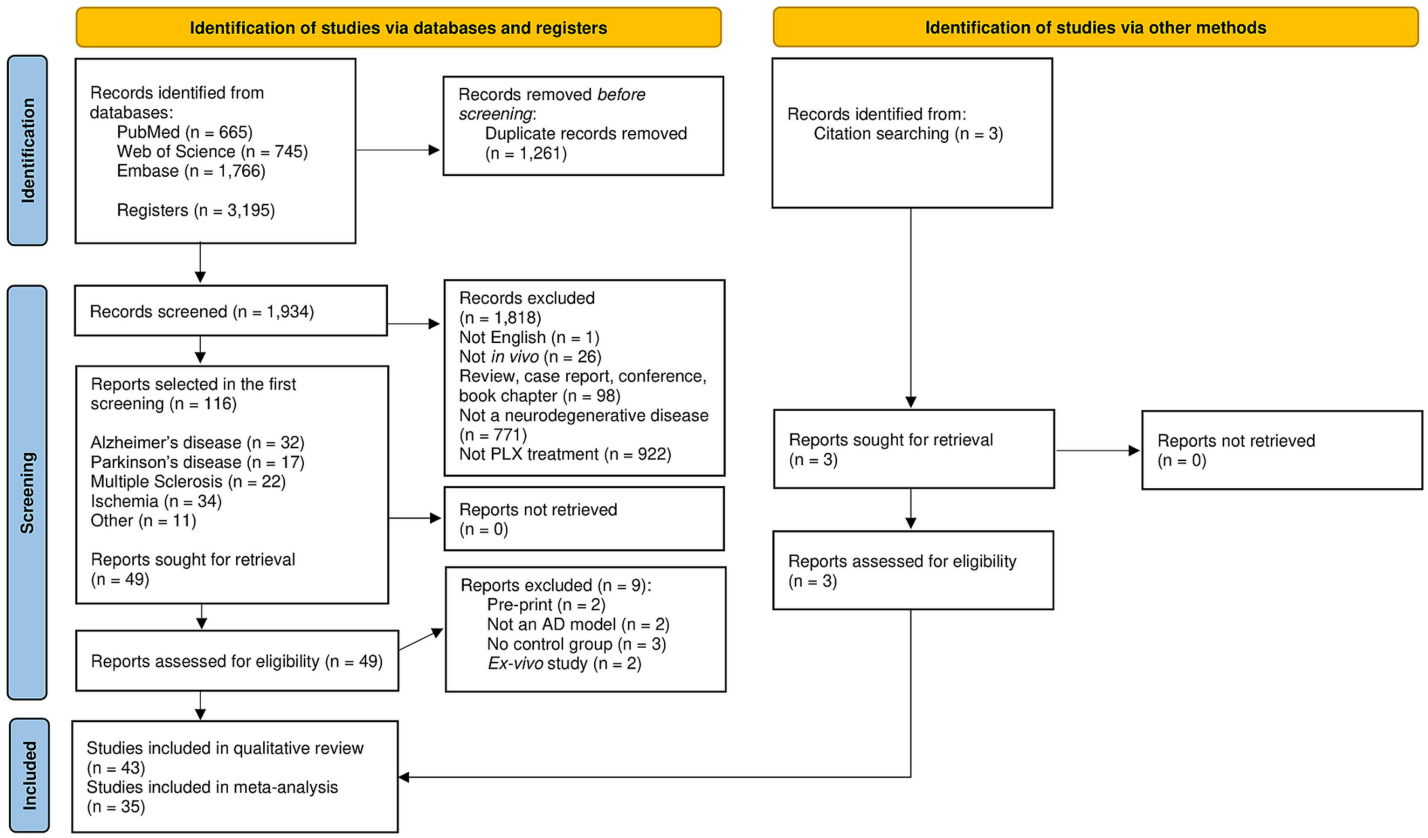
PRISMA flow diagram of the selection process.

A total of 3,195 articles were found in the initial search. After duplicate removal, 1,934 studies followed to title and abstract screening. The selected studies (*n* = 116) were classified according to the neurodegenerative disease model type: Ischemia (*n* = 34, 29.31%), Alzheimer’s disease (*n* = 32, 27.59%), Multiple Sclerosis (*n* = 22, 18.97%), Parkinson’s disease (*n* = 17, 14.66%), and other diseases (*n* = 11, 9.48%). Given the large number of studies identified, we decided to focus on a specific subgroup, as this approach was more feasible for conducting a systematic review. Therefore, as the two most common neurodegenerative diseases among elderly, the full-text studies with AD and PD models were retrieved. Only pre-clinical studies were found. Three studies were added from other sources (reference list from the manuscripts). After eligibility assessment, 17 (39.53%) studies with PD models (Yang et al., 2018; Dwyer et al., 2020; Liang et al., 2023; Pereira et al., 2023; Ma et al., 2024; Stoll et al., 2024; Thi Lai et al., 2024; Iba et al., 2025; Zhang et al., 2025; Oh et al., 2020; Jing et al., 2021; Li et al., 2021; Zhang et al., 2021; Abdel-Haq et al., 2022; Guo et al., 2022; Ruan et al., 2022; Bhatia et al., 2023) and 26 (60.47%) studies with AD models were selected for the qualitative analysis (Spangenberg et al., 2019; Dagher et al., 2015; Son et al., 2020; Benitez et al., 2021; Bennett et al., 2018; Delizannis et al., 2021; Tsai et al., 2020; Dodiya et al., 2021; Clayton et al., 2021; Lodder et al., 2021; Karaahmet et al., 2022; Wendt et al., 2022; Asai et al., 2015; Gaunt et al., 2023; Son et al., 2023; Weigel et al., 2023; Johnson et al., 2023; Wang et al., 2023; Kodali et al., 2024; Spangenberg et al., 2016; Sosna et al., 2018; Unger et al., 2018; Shi et al., 2019; Casali et al., 2020; Crapser et al., 2020; Michael et al., 2020). Figure 1 details the selection process, including the reasons for exclusion.

### 3.2 Quality assessment

**Figure 2:**
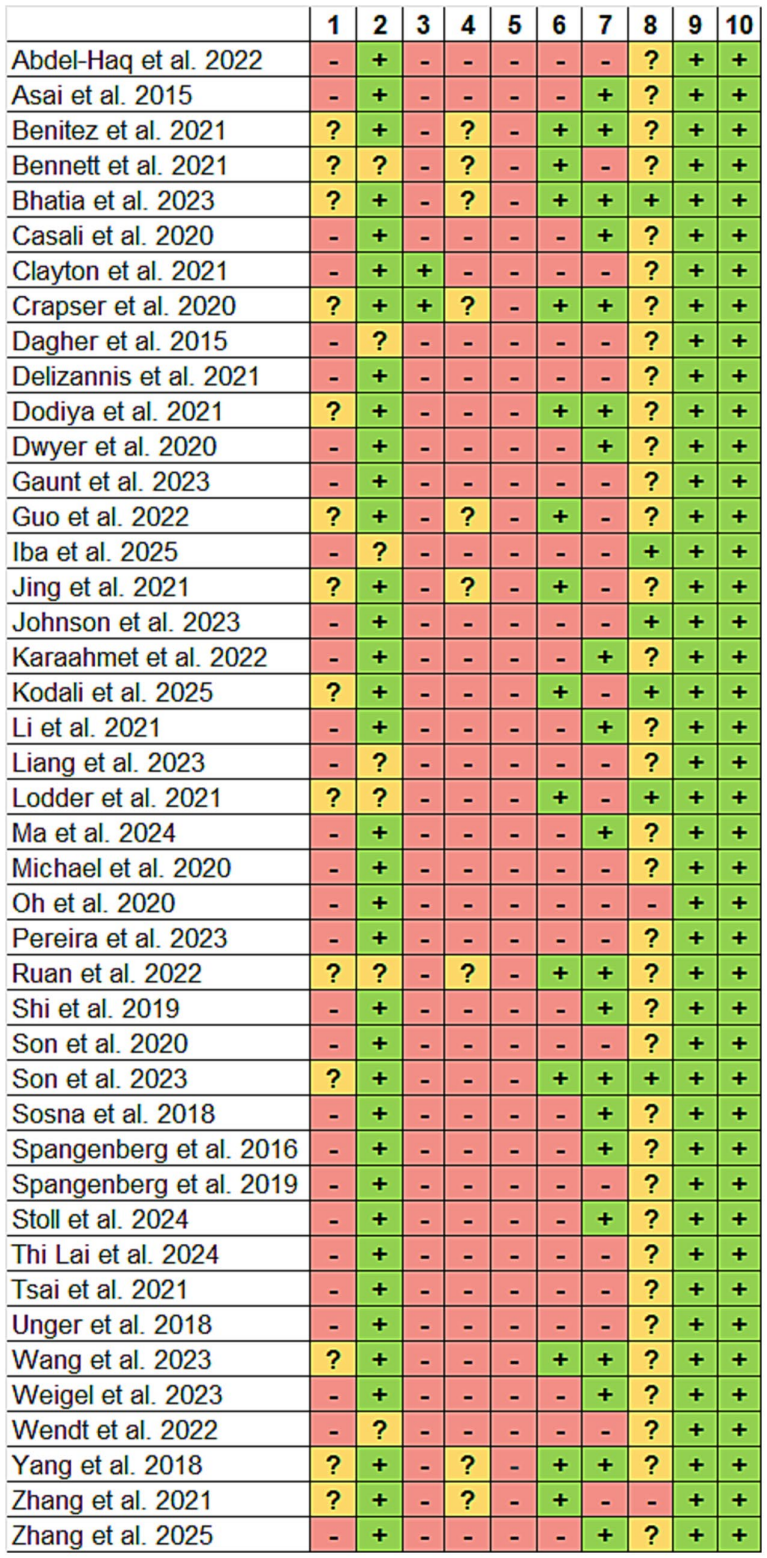
Methodological quality of included studies using the SYRCLE RoB tool. 1. Was the allocation sequence adequately generated and applied? (Selection bias); 2. Were the groups similar at baseline or were they adjusted for confounders in the analysis? (Selection bias); 3. Was the allocation adequately concealed? (Selection bias); 4. Were the animals randomly housed during the experiment? (Performance bias); 5. Were the caregivers and/or investigators blinded from knowledge which intervention each animal received during the experiment? (Performance bias); 6. Were animals selected at random for outcome assessment? (Detection bias); 7. Was the outcome assessor blinded? (Detection bias); 8. Were incomplete outcome data adequately addressed? (Attrition bias); 9. Are reports of the study free of selective outcome reporting? (Reporting bias); 10. Was the study apparently free of other problems that could result in high risk of bias? (Other). (?) Unclear; (+) Yes and (−) No.

The quality assessment of the studies is reported in Figure 2. The studies presented low risk of bias in the questions 2, 9, and 10, which refers to “selection bias - baseline characteristics”, “reporting bias - selective outcome reporting”, and “other sources of bias”, respectively. The third (selection bias - allocation concealment), fourth (performance bias - random housing), and fifth (performance bias - blinding) questions were the worst rated ones. Almost all studies showed unclear attribution bias as it was not clear if exclusions in the sample size were made (question 8). In addition, 14 studies (33%) reported randomization of groups and 20 studies (47%) reported blinding for the outcome assessment.

### 3.3 Characteristics of selected studies

#### 3.3.1 Overall characteristics of preclinical models

Nineteen rodent strains were used in PD studies: two studies used rats (11.76%; Sprague–Dawley, *n* = 1; Fischer 344, *n* = 1) (Oh et al., 2020; Stoll et al., 2024) and fifteen studies used mice (88.24%) (Yang et al., 2018; Dwyer et al., 2020; Pereira et al., 2023; Ma et al., 2024; Thi Lai et al., 2024; Iba et al., 2025; Zhang et al., 2025; Jing et al., 2021; Li et al., 2021; Zhang et al., 2021; Abdel-Haq et al., 2022; Guo et al., 2022; Ruan et al., 2022; Bhatia et al., 2023; Liang et al., 2023), predominantly C57BL/6 J (*n* = 11, 57.89%) (Yang et al., 2018; Jing et al., 2021; Zhang et al., 2025; Li et al., 2021; Zhang et al., 2021; Guo et al., 2022; Ruan et al., 2022; Liang et al., 2023; Pereira et al., 2023; Ma et al., 2024; Thi Lai et al., 2024). Other mouse models included Thy1-*α*-synuclein overexpression (*n* = 2, 10.53%) (Abdel-Haq et al., 2022; Iba et al., 2025), CD-1 (*n* = 1, 5.26%) (Bhatia et al., 2023), LRRK2 G2019S knock-in (*n* = 1, 5.26%) (Dwyer et al., 2020), myeloid-deficient RBP-J cKO (*n* = 1, 5.26%) (Liang et al., 2023), and Rag2^−/−^γc^−/−^ mice (*n* = 1, 5.26%) (Yang et al., 2018). Most studies used male animals (*n* = 14, 82.36%) (Yang et al., 2018; Dwyer et al., 2020; Stoll et al., 2024; Thi Lai et al., 2024; Zhang et al., 2025; Zarou et al., 2023; Oh et al., 2020; Jing et al., 2021; Li et al., 2021; Zhang et al., 2021; Abdel-Haq et al., 2022; Guo et al., 2022; Pereira et al., 2023; Ma et al., 2024), one study used both sexes (5.88%) (Bhatia et al., 2023), and two did not report sex (11.76%) (Liang et al., 2023; Iba et al., 2025). Animal ages ranged from 5–6 weeks to 18 months, with most studies using animals ≤2 months old (*n* = 7, 41.18%) (Yang et al., 2018; Oh et al., 2020; Jing et al., 2021; Zhang et al., 2021; Abdel-Haq et al., 2022; Guo et al., 2022; Zhang et al., 2025), while fewer used 2–3 months (*n* = 2, 11.76%) (Li et al., 2021; Thi Lai et al., 2024), 3 months (*n* = 3, 17.66%) (Dwyer et al., 2020; Pereira et al., 2023; Stoll et al., 2024), 5–9 months (*n* = 1, 5.88%) (Iba et al., 2025), >12 months (*n* = 2, 11.76%) (Bhatia et al., 2023; Ma et al., 2024), and two studies did not report age (11.76%) (Ruan et al., 2022; Liang et al., 2023).

The preclinical PD models in this review were mostly neurotoxin-based (*n* = 15, 88.24%) (Yang et al., 2018; Dwyer et al., 2020; Pereira et al., 2023; Ma et al., 2024; Stoll et al., 2024; Thi Lai et al., 2024; Zhang et al., 2025; Oh et al., 2020; Jing et al., 2021; Li et al., 2021; Zhang et al., 2021; Guo et al., 2022; Ruan et al., 2022; Bhatia et al., 2023; Liang et al., 2023), with only two using transgenic mice (Thy1-*α*-syn, 11.76%) (Abdel-Haq et al., 2022; Iba et al., 2025). Among neurotoxins, five studies used rotenone (29.41%) (Jing et al., 2021; Zhang et al., 2021; Guo et al., 2022; Ruan et al., 2022; Ma et al., 2024), three 1-methyl-4-phenyl-1,2,3,6-tetrahydropyridine (MPTP) (17.65%) (Yang et al., 2018; Li et al., 2021; Liang et al., 2023), two 6-hydroxydopamine (6-OHDA) (11.76%) (Oh et al., 2020; Pereira et al., 2023), one lipopolysaccharide (LPS) and paraquat (5.88%) (Dwyer et al., 2020), three α-synuclein pre-formed fibrils (PFF) (17.65%) (Bhatia et al., 2023; Stoll et al., 2024; Thi Lai et al., 2024), and three human α-synuclein induced expression (17.65%) (Abdel-Haq et al., 2022; Iba et al., 2025; Zhang et al., 2025). Rotenone was administered intraperitoneally (i.p.), mostly at 1.5 mg/kg daily for 3 weeks (Jing et al., 2021; Zhang et al., 2021; Guo et al., 2022; Ruan et al., 2022), with one study using 6.25 mg/kg daily, 5 days/week for 2–8 weeks (Ma et al., 2024). MPTP was administered as four i.p. injections, in the dose of 10 mg/kg (*n* = 1, 66.67%) (Li et al., 2021) and 20 mg/kg (*n* = 2, 33.33%) (Yang et al., 2018; Liang et al., 2023). 6-OHDA model was injected into the striatum (CPu) at 32 μg in rats (Oh et al., 2020) and 10 μg bilaterally in mice (Pereira et al., 2023). One study used the combination of 1 μg/μL of LPS, injected directly above the SNc, with 6 i.p. injections of paraquat (10 mg/kg), given every 48 h for 11 days (Dwyer et al., 2020). PFFs were injected into the left CPu in rats (16 μg) (Stoll et al., 2024) and either unilateral in right CPu (5 μg) (Thi Lai et al., 2024) or bilateral in anterior olfactory nuclei (Bhatia et al., 2023) in mice. Two studies used the Thy1-α-synuclein mice (Abdel-Haq et al., 2022; Iba et al., 2025) and one study used the rAAV-hSYN vector to express human α-synuclein in mice (Zhang et al., 2025) (the basic characteristics of PD selected studies are summarized in Table 1).

**Table 1:** Basic characteristics of Parkinson’s disease selected studies.

Parkinson’s disease					
Study		Strain	Sex/age	Model	
				Mutations/neurotoxin	Administration route, dose and frequency
Yang et al.	2018	C57BL/6 L and Rag2−/−γc/− mice (Rag2/Il2rg compound mutant mice lacking T, B, NK, and NKT cells)	Male/7-8-wk-old	MPTP	4 injections of 20 mg/kg, i.p.
Dwyer et al.	2020	LRRK2 G2019S knock-in mice	Male/3-mo-old	LPS + Paraquat	LPS: 1 μg/μL, directly above the SNc Paraquat: 6 injections of 10 mg/kg, i.p., every 48 h for 11 days
Oh et al.	2020	Sprague–Dawley rats	Male/56-days-old	6-OHDA	32 μg into right CPu
Jing et al.	2021	C57BL/6 J mice	Male/8-wk-old	Rotenone	1.5 mg/kg, i.p., daily for 3 weeks
Li et al.	2021	C57BL/6 J mice	Male/10-wk-old	MPTP	4 injections of 10 mg/kg, i.p.
Zhang et al. a	2021	C57BL/6 J mice	Male/8-wk-old	Rotenone	1.5 mg/kg, i.p., daily for 3 weeks
Abdel-Haq et al.	2022	Thy1-α-syn mice	Male/5-6-wk-old	Human α-syn expression	NA
Guo et al.	2022	C57BL/6 J mice	Male/8-wk-old	Rotenone	1.5 mg/kg, i.p., daily for 3 weeks
Ruan et al.	2022	C57BL/6 J mice	Male/NR	Rotenone	1.5 mg/kg, i.p., daily for 3 weeks
Bhatia et al.	2023	CD-1 mice	Male and female/18-mo-old	α-syn PFF	5 μg/ 1 μL/ bilaterally into anterior olfactory nuclei
Liang et al.	2023	C57BL/6 J and RBP-J cKO mice	NR	MPTP	4 injections of 20 mg/kg, i.p.
Pereira et al.	2023	C57BL/6 J mice	Male/3-mo-old	6-OHDA	10 μg bilaterally into CPu
Ma et al.	2024	C57BL/6 J mice	Male/12-mo-old	Rotenone	6.25 mg/kg daily, 5 days per week for 2 wk. or 8 wk
Stoll et al.	2024	Fischer 344 rats	Male/3-mo-old	α-syn PFF	16 μg into left CPu
Thi Lai et al.	2024	C57BL/6 J mice	Male/10-wk-old	α-syn PFF	5 μg into right CPu
Iba et al.	2025	Thy1-α-syn mice	NR/ 5-7-mo-old and 7-9-mo-old	Human α-syn expression	NA
Zhang et al.	2025	C57BL/6 J mice	Male/8-wk-old	Human syn overexpression	rAAV-hSYN vector injection

6-OHDA, 6-hydroxydopamine; CPu, striatum; d, days or days; i.p., intraperitoneal; LPS, lipopolysaccharide; mo, month or months; MTPT, 1-methyl-4-phenyl-1,2,3,6-tetrahydropyridine; NA, not applicable; NR, not reported; syn, synuclein; wk, week; wks, weeks.

Thirty mouse strains were used in AD studies, all involving genetic models, with five studies combining genetic models with intracerebral tau administration (pre-formed fibrils or AAV2/6-SYN1-P301L tau) (Asai et al., 2015; Delizannis et al., 2021; Clayton et al., 2021; Lodder et al., 2021; Johnson et al., 2023). The most common genetic models were 5xFAD (*n* = 12, 40%) (Spangenberg et al., 2019; Spangenberg et al., 2016; Wang et al., 2023; Kodali et al., 2024; Sosna et al., 2018; Casali et al., 2020; Crapser et al., 2020; Son et al., 2020; Delizannis et al., 2021; Tsai et al., 2020; Wendt et al., 2022; Son et al., 2023), 3xTg (*n* = 4, 13.33%) (Dagher et al., 2015; Crapser et al., 2020; Karaahmet et al., 2022; Weigel et al., 2023), and APP/PS1 (*n* = 4, 13.33%) (Unger et al., 2018; Michael et al., 2020; Dodiya et al., 2021; Karaahmet et al., 2022). Most studies included both sexes (*n* = 14, 53.85%) (Spangenberg et al., 2019; Spangenberg et al., 2016; Clayton et al., 2021; Karaahmet et al., 2022; Johnson et al., 2023; Wang et al., 2023; Sosna et al., 2018; Unger et al., 2018; Casali et al., 2020; Crapser et al., 2020; Michael et al., 2020; Bennett et al., 2018; Delizannis et al., 2021; Tsai et al., 2020), five used only males (19.23%) (Asai et al., 2015; Shi et al., 2019; Benitez et al., 2021; Dodiya et al., 2021; Gaunt et al., 2023), four only females (15.38%) (Son et al., 2020; Son et al., 2023; Weigel et al., 2023; Kodali et al., 2024), and three did not report sex (11.54%) (Dagher et al., 2015; Lodder et al., 2021; Wendt et al., 2022). Animal ages ranged from 24 days to 21–22 months, with eight studies using <2 months (26.67%) (Dagher et al., 2015; Spangenberg et al., 2016; Crapser et al., 2020; Benitez et al., 2021; Delizannis et al., 2021; Dodiya et al., 2021; Lodder et al., 2021; Wendt et al., 2022), eight using 3–4 months (26.67%) (Asai et al., 2015; Casali et al., 2020; Tsai et al., 2020; Clayton et al., 2021; Lodder et al., 2021; Gaunt et al., 2023; Wang et al., 2023; Kodali et al., 2024), two using 6–7 months (6.67%) (Shi et al., 2019; Benitez et al., 2021), four using 8–10 months (13.33%) (Spangenberg et al., 2016; Son et al., 2020; Wendt et al., 2022; Son et al., 2023), and eight using >12 months (26.67%) (Dagher et al., 2015; Spangenberg et al., 2016; Unger et al., 2018; Crapser et al., 2020; Michael et al., 2020; Bennett et al., 2018; Karaahmet et al., 2022; Weigel et al., 2023) (the basic characteristics of AD selected studies are summarized in Table 2).

**Table 2:** Basic characteristics of Alzheimer’s disease selected studies.

Alzheimer’s disease					
Study		Strain	Sex/age	Model	
				Mutations/neurotoxin	Administration route, dose and frequency
Dagher et al.	2015	3xTg	NR/13-mo-old	NA	NA
Asai et al	2015	C57BL/6 and Tau P301S	Male/3.5-mo-old	AAV2/6-SYN1-P301L tau	1 μl into medial entorhinal cortex, unilaterally
Spangenberg et al.	2016	CSF1R-iCRE/Rosa26YFP and 5xFAD	Male and female/1.5, 2, 10, 14-mo-old	NA	NA
Sosna et al.	2018	5xFAD	Male and female/2-mo-old	NA	NA
Unger et al.	2018	APP/PS1	Female and male/12-mo-old	NA	NA
Spangenberg et al.	2019	5xFAD	Female and male/1.5-mo-old	NA	NA
Shi	2019	P301S crossed to APOE4 KI (TE4) or Apoe KO (TEKO)	Male/6-mo-old	NA	NA
Casali et al.	2020	5xFAD	Male and female/4-mo-old	NA	NA
Crapser et al.	2020	5xFAD and 3xTg-AD	Male and female/5xFAD: 1.5 mo-old; 3xTg: 17 mo-old	NA	NA
Michael et al.	2020	APP/PS1	Female and male/12-mo-old	NA	NA
Son et al.	2020	5xFAD	Female/9-mo-old	NA	NA
Benitez et al.	2021	AppNL-F and AppNL-G-F	Male/AppNL-G-F: 1.5-mo-old; AppNL-F: 7 mo-old	NA	NA
Bennett et al.	2021	Tg4510	Males and females/12-mo-old	NA	NA
Delizannis et al.	2021	5xFAD	Males and females/1.5-mo-old	AD brain-derived pathological tau	2.5 μL of 0.4 mg/mg into right hippocampus and overlying cortex of 3-mo-old mice
Tsai et al.	2021	5xFAD	Male and female/4-mo-old	NA	NA
Dodiya et al.	2021	APP/PS1	Male/24-days-old	NA	NA
Clayton et al.	2021	AppNL-G-F	Male and female/4-mo-old	AAV2/6-SYN1-P301L tau	Nine tenths of 1 μL at a viral titer of 1.2 × 10^11^ into medial entorhinal cortex
Lodder et al.	2021	5xFAD crossed to Tau P301S	NR/4-mo-old	tau K18 P301L fibrils	5 μL of 333 μM into hippocampus and frontal cortex, unilateral
Karaahmet et al.	2022	APP/PS1 and 3xTg	Male and female/APP/PS1:14-mo-old; Male/3xTg: 21–22-mo-old	NA	NA
Wendt et al.	2022	5xFAD	NR/9-mo-old	NA	NA
Gaunt et al.	2023	AppNL-G-F	Male/4-mo-old	NA	NA
Son et al.	2023	5xFAD	Female/8-mo-old	NA	NA
Weigel et al.	2023	3xTg	Female/13-mo-old	NA	NA
Johnson et al.	2023	Tg2541	Male and female/2-mo-old	tau K18 P301L fibrils	10 μL of 1.5 mg/mL into hippocampus and overlying cortex (forebrain), unilateral 10 μl of 1.5 mg/ml into midbrain, bilateral
Wang et al.	2023	5xFAD	Male and female/4-mo-old	NA	NA
Kodali et al.	2025	5xFAD	Female/3-mo-old	NA	NA

APP, amyloid precursor protein; d, days or days; mo, month or months; NA, not applicable; NR, not reported.

#### 3.3.2 Overall characteristics of treatment

It was noticed that half of the studies used PLX3397 (*n* = 24, 52.17%) (Spangenberg et al., 2019; Yang et al., 2018; Iba et al., 2025; Asai et al., 2015; Spangenberg et al., 2016; Sosna et al., 2018; Shi et al., 2019; Son et al., 2020; Bennett et al., 2018; Delizannis et al., 2021; Lodder et al., 2021; Wendt et al., 2022; Dwyer et al., 2020; Son et al., 2023; Weigel et al., 2023; Johnson et al., 2023; Wang et al., 2023; Oh et al., 2020; Jing et al., 2021; Li et al., 2021; Zhang et al., 2021; Guo et al., 2022; Ruan et al., 2022; Stoll et al., 2024) and the other half PLX5622 (*n* = 22, 47.83%) (Spangenberg et al., 2019; Abdel-Haq et al., 2022; Unger et al., 2018; Casali et al., 2020; Crapser et al., 2020; Michael et al., 2020; Benitez et al., 2021; Tsai et al., 2020; Dodiya et al., 2021; Clayton et al., 2021; Karaahmet et al., 2022; Gaunt et al., 2023; Bhatia et al., 2023; Johnson et al., 2023; Kodali et al., 2024; Liang et al., 2023; Pereira et al., 2023; Ma et al., 2024; Thi Lai et al., 2024; Zhang et al., 2025; Dagher et al., 2015; Spangenberg et al., 2016). A similar proportion is observed when we split the studies by type of disease, with PLX3397 and PLX5622 used by 58.82% (*n* = 10) (Yang et al., 2018; Dwyer et al., 2020; Oh et al., 2020; Jing et al., 2021; Li et al., 2021; Zhang et al., 2021; Guo et al., 2022; Ruan et al., 2022; Stoll et al., 2024; Iba et al., 2025) and 41.18% (*n* = 7) (Abdel-Haq et al., 2022; Bhatia et al., 2023; Liang et al., 2023; Pereira et al., 2023; Ma et al., 2024; Thi Lai et al., 2024; Zhang et al., 2025), respectively, in PD studies; in AD, PLX3397 was used by 45.83% (*n* = 11) of the studies (Asai et al., 2015; Sosna et al., 2018; Wang et al., 2023; Shi et al., 2019; Son et al., 2020; Bennett et al., 2018; Delizannis et al., 2021; Lodder et al., 2021; Wendt et al., 2022; Son et al., 2023; Weigel et al., 2023), PLX5622 was used by 50.00% (*n* = 12) (Dagher et al., 2015; Unger et al., 2018; Gaunt et al., 2023; Kodali et al., 2024; Casali et al., 2020; Crapser et al., 2020; Michael et al., 2020; Benitez et al., 2021; Tsai et al., 2020; Dodiya et al., 2021; Clayton et al., 2021; Karaahmet et al., 2022), and three studies (4.17%) used both (Spangenberg et al., 2019; Spangenberg et al., 2016; Johnson et al., 2023). Treatments were mostly oral, either in chow (*n* = 34, 79.07%) (Spangenberg et al., 2019; Yang et al., 2018; Zhang et al., 2025; Dagher et al., 2015; Asai et al., 2015; Spangenberg et al., 2016; Sosna et al., 2018; Unger et al., 2018; Shi et al., 2019; Casali et al., 2020; Crapser et al., 2020; Michael et al., 2020; Dwyer et al., 2020; Benitez et al., 2021; Bennett et al., 2018; Delizannis et al., 2021; Tsai et al., 2020; Dodiya et al., 2021; Clayton et al., 2021; Lodder et al., 2021; Karaahmet et al., 2022; Wendt et al., 2022; Gaunt et al., 2023; Li et al., 2021; Weigel et al., 2023; Johnson et al., 2023; Wang et al., 2023; Kodali et al., 2024; Abdel-Haq et al., 2022; Bhatia et al., 2023; Liang et al., 2023; Pereira et al., 2023; Ma et al., 2024; Stoll et al., 2024) or gavage (*n* = 8, 18.60%) (Oh et al., 2020; Jing et al., 2021; Zhang et al., 2021; Guo et al., 2022; Ruan et al., 2022; Thi Lai et al., 2024; Son et al., 2020; Son et al., 2023), except one PD study combining chow and i.p. injections (Iba et al., 2025). One study did not report the route (Yang et al., 2018). In PD, 10 studies used PLX chow (58.82%) (Dwyer et al., 2020; Li et al., 2021; Abdel-Haq et al., 2022; Bhatia et al., 2023; Liang et al., 2023; Pereira et al., 2023; Ma et al., 2024; Stoll et al., 2024; Iba et al., 2025; Zhang et al., 2025) and six used gavage (35.29%) (Oh et al., 2020; Jing et al., 2021; Zhang et al., 2021; Guo et al., 2022; Ruan et al., 2022; Thi Lai et al., 2024). In AD, 24 studies used chow (92.31%) (Spangenberg et al., 2019; Dagher et al., 2015; Benitez et al., 2021; Bennett et al., 2018; Delizannis et al., 2021; Tsai et al., 2020; Dodiya et al., 2021; Clayton et al., 2021; Lodder et al., 2021; Karaahmet et al., 2022; Wendt et al., 2022; Gaunt et al., 2023; Asai et al., 2015; Weigel et al., 2023; Johnson et al., 2023; Wang et al., 2023; Kodali et al., 2024; Spangenberg et al., 2016; Sosna et al., 2018; Unger et al., 2018; Shi et al., 2019; Casali et al., 2020; Crapser et al., 2020; Michael et al., 2020) and two gavage (7.69%) (Son et al., 2020; Son et al., 2023). PLX5622 was mainly administered at 1200 mg/kg chow (*n* = 21) (Spangenberg et al., 2019; Abdel-Haq et al., 2022; Casali et al., 2020; Crapser et al., 2020; Michael et al., 2020; Benitez et al., 2021; Tsai et al., 2020; Dodiya et al., 2021; Clayton et al., 2021; Karaahmet et al., 2022; Gaunt et al., 2023; Johnson et al., 2023; Bhatia et al., 2023; Kodali et al., 2024; Liang et al., 2023; Pereira et al., 2023; Ma et al., 2024; Zhang et al., 2025; Dagher et al., 2015; Spangenberg et al., 2016; Unger et al., 2018), with two studies using 300 mg/kg (Dagher et al., 2015; Benitez et al., 2021) and one gavage at 65 mg/kg (Thi Lai et al., 2024). PLX3397 doses varied: PD studies used 290–600 mg/kg in chow (Li et al., 2021; Iba et al., 2025) or 30–40 mg/kg by gavage (Yang et al., 2018; Oh et al., 2020; Jing et al., 2021; Zhang et al., 2021; Guo et al., 2022; Ruan et al., 2022) and AD studies used 275–1,000 mg/kg in chow (Spangenberg et al., 2019; Dagher et al., 2015; Wendt et al., 2022; Weigel et al., 2023; Johnson et al., 2023; Wang et al., 2023; Asai et al., 2015; Spangenberg et al., 2016; Sosna et al., 2018; Shi et al., 2019; Benitez et al., 2021; Bennett et al., 2018; Delizannis et al., 2021; Lodder et al., 2021) or 50 mg/kg by gavage (Son et al., 2020; Son et al., 2023) (the treatment protocols in the selected PD and AD studies are summarized in Table 3).

**Table 3:** Characteristics of PLX-treatment protocol used by the selected studies.

Study		Treatment			% Microglia reduction
		PLX type	Route/dose/duration	Microglia removal time	
Parkinson’s disease					
Yang et al.	2018	PLX3397	NR, 40 mg/kg daily for 28 d	Started 21 d before + 7 d after model induction *(Before and during)*	Flow cytometry: ~90% reduction of microglia (CD11b + CD45int) CD11b + CD45 high
Dwyer et al.	2020	PLX3397	Rodent chow for 26 d	Started 14 d before + 12d after model induction *(Before and during)*	NR
Oh et al.	2020	PLX3397	Oral gavage, 30 mg/kg daily for 30 d	From d 7 to d 28 after surgery *(During)*	NR
Jing et al.	2021	PLX3397	Oral gavage, 40 mg/kg/d, for 4 wk	Started 7 d before + 21 d after model induction *(Before and during)*	90% of Iba-1 counts in the LC of control group 70% of Iba-1 counts in the LC of PD group
Li et al.	2021	PLX3397	Rodent chow, 290 mg/kg, for 22, 24, 26, 28, 35 d *Repopulation 1:* regular chow at the d of PD induction *Repopulation 2:* PD was induced after 7 d of withdrawn + more 7 d	Started 21 d before + 1, 3, 5, 7 or 14 d after model induction *(Before and during)*	30% of Iba-1 counts in the SNc after 7 d 90% of Iba-1 counts in the SNc after 21 d
Zhang et al.	2021	PLX3397	Oral gavage, 40 mg/kg daily for 7 d and every 48 h for more 28 d	Started 7 d before + 27 d after model induction *(Before and during)*	NR
Guo et al.	2022	PLX3397	Oral gavage, 40 mg/kg daily for 7 d and every 48 h for more 21 d	Started 7 d before + 21 d after model induction *(Before and during)*	NR
Ruan et al.	2022	PLX3397	Oral gavage, 40 mg/kg daily for 7 d and every 48 h for more 21 d	Started 7 d before + 21 d after model induction *(Before and during)*	~85% of Iba-1 counts in the SN*
Stoll et al.	2024	PLX3397	Rodent chow, 600 mg/kg, for 60 or 180 d	Started on the day of model induction + 60 or 180 d after *(During and after)* Other group started 7 d before + 60 d after model induction *(Before and during)*	45% of Iba-1 counts in control mice and 36.6% in PD mice after 2 mo of treatment 56% of Iba-1 counts in control mice and 36% in PD mice after 6 mo of treatment
Iba et al.	2025	PLX3397	Rodent chow, 290 mg/kg, and 40 mg/kg i.p. twice per wk. for 3 wk	*(During)*	~88% of Iba-1 counts in the cortex, HPC, and CPu*
Abdel-Haq et al.	2022	PLX5622	Rodent chow, 1,200 mg/kg, for 16–17 wk	*(During)*	~80% of Iba-1 counts in the cerebellum ~ 65% of Iba-1 counts in the SN ~ 75% of Iba-1 counts in the CPu
Bhatia et al.	2023	PLX5622	Rodent chow, 1,200 mg/kg, for 2 wk. + withdrawn for 7 d	Started 7 d before + 7 d after + 7 d withdraw *(Before and during)*	Flow cytometry: 60% reduction of microglia after 2 wk. (CD45 + CD11b + F4/80+)
Liang et al.	2023	PLX5622	Rodent chow, 1,200 mg/kg, for 14 d	Started 7 d before + 7 d after model induction *(Before and after)*	Flow cytometry and FACS: ~77% after 7 d (CD11b + CD45low) whole brain* ~ 72% of Iba-1 counts after 7 d*
Pereira et al.	2023	PLX5622	Rodent chow, 1,200 mg/kg, for 31 d	Started 14 d before + 17 d after model induction *(Before and during)*	∼90% Iba1 counts in control mice and ∼80% in the PD group
Ma et al.	2024	PLX5622	Rodent chow, 1,200 mg/kg, for 3 or 9 wk	Started 7 ds before plus 2 wk. or 8 wk. during model induction *(Before and during)*	83.4% of Iba-1 counts after 1 wk. in the SN
Thi Lai et al.	2024	PLX5622	Oral gavage, 65 mg/kg daily for 3, 4, or 14 wk	Started 2 wk. before + 1, 2, or 12 wk. after model induction *(Before and during)*	47.95% decrease in Iba-1 in whole brain (WB)
Zhang et al.	2025	PLX5622	Rodent chow, 1,200 mg/kg, for 11 wk	Started 3 wk. before + 8 wk. after model induction *(Before and during)*	92.5% of Iba-1 counts
Alzheimer’s disease					
Asai et al	2015	PLX3397	Rodent chow, 290 mg/kg, for 1 mo before injection + 1 mo after (injected animals) and 4 wk. (PS19 mice)	Before and during	86% of Iba1 counts in the granule cell layer of the dentate gyrus (for injected animals) > 90% of Iba1 counts in the entorhinal cortex and dentate gyrus (for PS19 mice)
Sosna et al.	2018	PLX3397	Rodent chow, 290 mg/kg, for 3 mo	During	~70–80% of Iba-1 counts in the cortex, HPC, and amygdala of AD group
Shi	2019	PLX3397	Rodent chow, 400 mg/kg, for 3 mo	During	∼90% of Iba1 area in the HPC after 7 d and virtually all microglia after 21 d
Son et al.	2020	PLX3397	Oral gavage; 50 mg/kg for 30 ds	After	42% of Iba-1 counts in the cortex of AD group
Bennett et al.	2021	PLX3397	Rodent chow, 290 mg/kg, for 3 mo	After	30% of Iba-1 counts in the cortex of AD group
Delizannis et al.	2021	PLX3397	Rodent chow, 1,000 mg/kg, for 1 wk. + 290 mg/kg for 5 wk. + (injections of Tau) + 290 mg/kg for 3 mo (18 weeks in total)	Before and during	92% of Iba-1 integrated density in the cortex and 62% in the subiculum of AD group
Lodder et al.	2021	PLX3397	Rodent chow, 1,000 mg/kg, for 1.5 mo	1.5 mo after PFF injections	81 ± 6% of Iba1 area in the cortex
Wendt et al.	2022	PLX3397	Rodent chow, 290 mg/kg, for 8 wk	After	~60% of Iba-1 counts in the cortex in AD group and ~80% in control
Son et al.	2023	PLX3397	Oral gavage, 50 mg/kg, for 1 mo	After	NR
Weigel et al.	2023	PLX3397	Rodent chow, 660 mg/kg, for 14 d	After	>98% in Iba-1 counts in the ventromedial hypothalamus in both AD and control group
Wang et al.	2023	PLX3397	*Repopulation*: Rodent chow, 290 mg/kg, for 1 mo + withdraw for 1 mo, 3 mo, or 8 mo	During	50%* in Iba1 counts in the HPC of AD group
Dagher et al.	2015	PLX5622	Rodent chow, 300 mg/kg and 1,200 mg/kg, for 7 or 21 d for the control mice Rodent chow, 300 mg/kg, for 6 wk. or 3 mo for the AD mice	After	~30% Iba1 counts after 7 and 21 d of treatment with 300 mg/kg in the control mice 80% Iba1 counts after 7 d of treatment with 1,200 mg/kg in the control mice 35% Iba1 counts after 3 mo of treatment with 300 mg/kg in the AD group (HPC, thalamus, and subiculum)
Unger et al.	2018	PLX5622	Rodent chow, 1,200 mg/kg, for 28 d	After	82.35% of Iba-1 counts in the HPC and 92.12% in cortex of control group 70.04% of Iba-1 counts in the HPC and 67.77% in cortex of AD group
Casali et al.	2020	PLX5622	Rodent chow, 1,200 mg/kg, for 28 d + withdraw for 28 d	During	>50% of Iba-1 counts in the brain (30% in the subiculum, 50% in the HPC, and 70% in cortex and thalamus)
Crapser et al.	2020	PLX5622	Rodent chow, 1,200 mg/kg, for 10 wk. (5xFAD) and 1 mo (3xTg)	5xFAD: before and during; 3xTg: after	84%* of Iba-1 counts in the subiculum and 96%* in the cortex of control mice 59%* of Iba-1 in the subiculum and 97%* in the cortex of 5xFAD mice 60%* of Iba-1 in the subiculum and 83%* in the cortex of 3xTg mice
Michael et al.	2020	PLX5622	Rodent chow, 1,200 mg/kg, for 28 d	After	82.35%* of Iba-1 counts in the HPC of control group and 70.04%* of AD group 92.11%* of Iba-1 counts in the cortex of control group and 67.77%* of AD group
Benitez et al.	2021	PLX5622	Rodent chow, 300 mg/kg and 1,200 mg/kg, for 3 mo (AppNL-F) or 2 mo (AppNL-G-F)	Before and during	52.80%* Iba1 density in the HPC of control mice (300 mg/kg) 97.78%* Iba1 density in the HPC of control mice (1,200 mg/kg) 48.21%* Iba1 density in the HPC of 3.5 mo-old AppNL-G-F mice (300 mg/kg) 96.82%* Iba1 density in the HPC of 3.5 mo-old ApppNL-G-F mice (1,200 mg/kg) 49.98%* Iba1 density in the HPC of 10 mo-old AppNL-F mice (300 mg/kg) 85.98%* Iba1 density in the HPC of 10 mo-old AppNL-F mice (1,200 mg/kg)
Tsai et al.	2021	PLX5622	Rodent chow, 1,200 mg/kg, for 28 d *Repopulation*: rodent chow, 1,200 mg/kg, for 28 d + withdraw for 28 d	After	NR
Dodiya et al.	2021	PLX5622	Rodent chow, 1,200 mg/kg, for 9 wk. or 3 mo	Before	>98% of Iba-1 counts in the cerebral cortex of AD mice treated for 9 wk
Clayton et al.	2021	PLX5622	Rodent chow, 1,200 mg/kg, for 2 mo (1 mo prior Tau injections)	Before and during (for Tau) During	>93% of Iba1 area in the cortex of AD and control groups
Karaahmet et al.	2022	PLX5622	*Repopulation*: Rodent chow, 1,200 mg/kg, for 2 wk. + withdraw for 1 mo	During and after	50% decrease in total microglia coverage in 3xTg brains and 65% decrease in APP/PS1 brains
Gaunt et al.	2023	PLX5622	Rodent chow, 1,200 mg/kg, for 60 d	During	82% in Iba-1 area coverage in the entorhinal cortex and 70% in the deep cerebellar nuclei
Kodali et al.	2025	PLX5622	Rodent chow, 1,200 mg/kg, for 10 d	During	65% Iba1 counts in the HPC and cortex of AD group
Spangenberg et al.	2016	PLX3397 and PLX5622	Rodent chow, 600 mg/kg, for 7 or 28 d (PLX3397) Rodent chow, 1,200 mg/kg, for 28 d (PLX5622)	Before, during, and after	∼88% of CSF1R and ∼99% of Iba-1 counts in the cortex of 2-mo-old CSF1R-iCRE/Rosa26YFP mice after 7 d of treatment (PLX3397) ∼ 80% of Iba-1 counts in the HPC, cortex, and thalamus of 10 mo-old 5xFAD mice after 28 d of treatment (PLX3397) ∼ 95% of Iba-1 counts in the HPC, cortex, and thalamus of 1.5 mo-old 5xFAD mice after 28 d of treatment (PLX3397)
Spangenberg et al.	2019	PLX3397 and PLX5622	Rodent chow, 1,200 mg/kg, for 10 or 24 wk. (PLX5622) Rodent chow, 600 mg/kg, for 14 wk. (PLX3397) *Repopulation:* rodent chow, 1,200 mg/kg, for 10 wk. + 1 mo withdraw	Before and during	90% of Iba-1 counts after 5 d of treatment and 97–100% of Iba-1 counts after 24 wk. of treatment (PLX5622)
Johnson et al.	2023	PLX3397 and PLX5622	Rodent chow, 275 mg/kg (PLX3397) and 1,200 mg/kg (PLX5622) for 2 mo (acute), 5 mo (chronic), and until death Rodent chow, 275 mg/kg (PLX3397), for 3 mo (interventional treatment) and 5 mo (intermittently, 3 wk. on - 3 wk. off)	During and after	~60% area of Iba1 and P2yr12 in both the forebrains and hindbrains of AD group and control

AD, Alzheimer’s disease; CPu, striatum; d, day or days; HPC, hippocampus; LC, locus coeruleus; mo, month or months; NA, not applicable; NR, not reported; PD: Parkinson’s disease; SN, substantia nigra; SNc, substantia nigra pars compacta; wk, week or weeks.

There was a large variation in the duration of microglial depletion across studies. The most frequently used PLX treatment lasted ~30 days (*n* = 23). In PD studies, most depleted microglia for 21–35 days (*n* = 12, 60.00%) (Yang et al., 2018; Dwyer et al., 2020; Thi Lai et al., 2024; Iba et al., 2025; Oh et al., 2020; Jing et al., 2021; Li et al., 2021; Zhang et al., 2021; Guo et al., 2022; Ruan et al., 2022; Pereira et al., 2023; Ma et al., 2024), with shorter (14 days, *n* = 2, 10%) (Bhatia et al., 2023; Liang et al., 2023) or longer (60–180 days, *n* = 5) (Abdel-Haq et al., 2022; Ma et al., 2024; Stoll et al., 2024; Thi Lai et al., 2024; Zhang et al., 2025) durations less common. In AD studies, depletion typically lasted around one month (*n* = 10, 29.41%) (Asai et al., 2015; Spangenberg et al., 2016; Unger et al., 2018; Casali et al., 2020; Crapser et al., 2020; Michael et al., 2020; Son et al., 2020; Tsai et al., 2020; Son et al., 2023; Wang et al., 2023) or between 1–2 months (*n* = 10, 29.41%) (Spangenberg et al., 2019; Dagher et al., 2015; Crapser et al., 2020; Benitez et al., 2021; Dodiya et al., 2021; Clayton et al., 2021; Lodder et al., 2021; Wendt et al., 2022; Gaunt et al., 2023; Johnson et al., 2023), with some studies using 3–5 months (*n* = 8, 23.53%) (Spangenberg et al., 2019; Dagher et al., 2015; Unger et al., 2018; Shi et al., 2019; Benitez et al., 2021; Delizannis et al., 2021; Dodiya et al., 2021; Johnson et al., 2023), less than 1 month (*n* = 4, 11.76%) (Dagher et al., 2015; Spangenberg et al., 2016; Weigel et al., 2023; Kodali et al., 2024), and 5 or more months (*n* = 2, 5.88%) (Spangenberg et al., 2019; Johnson et al., 2023). Most PD studies began depletion before and continued during model induction/ progression (*n* = 14) (Yang et al., 2018; Dwyer et al., 2020; Pereira et al., 2023; Ma et al., 2024; Thi Lai et al., 2024; Zhang et al., 2025; Jing et al., 2021; Li et al., 2021; Zhang et al., 2021; Abdel-Haq et al., 2022; Guo et al., 2022; Ruan et al., 2022; Bhatia et al., 2023; Liang et al., 2023), whereas only three studies started PLX-treatment during the model progression (Oh et al., 2020; Stoll et al., 2024; Iba et al., 2025). In AD studies, depletion usually started after main pathological hallmarks were established (*n* = 11) (Dagher et al., 2015; Unger et al., 2018; Weigel et al., 2023; Crapser et al., 2020; Michael et al., 2020; Son et al., 2020; Bennett et al., 2018; Tsai et al., 2020; Lodder et al., 2021; Wendt et al., 2022; Son et al., 2023), followed by during (*n* = 7) (Sosna et al., 2018; Shi et al., 2019; Casali et al., 2020; Clayton et al., 2021; Gaunt et al., 2023; Wang et al., 2023; Kodali et al., 2024), before and during (*n* = 6) (Spangenberg et al., 2019; Asai et al., 2015; Crapser et al., 2020; Benitez et al., 2021; Delizannis et al., 2021; Clayton et al., 2021), during and after (*n* = 2) (Karaahmet et al., 2022; Johnson et al., 2023), before (*n* = 1) (Dodiya et al., 2021), and before, during, and after (*n* = 1) (Spangenberg et al., 2016). In addition, some studies employed the repopulation paradigm, in which animals return to regular chow diet after microglial depletion to allow for the repopulation by these cells. This approach was performed by two PD model studies (Li et al., 2021; Bhatia et al., 2023) and five AD model studies (Spangenberg et al., 2019; Casali et al., 2020; Tsai et al., 2020; Karaahmet et al., 2022; Wang et al., 2023).

**Figure 3:**
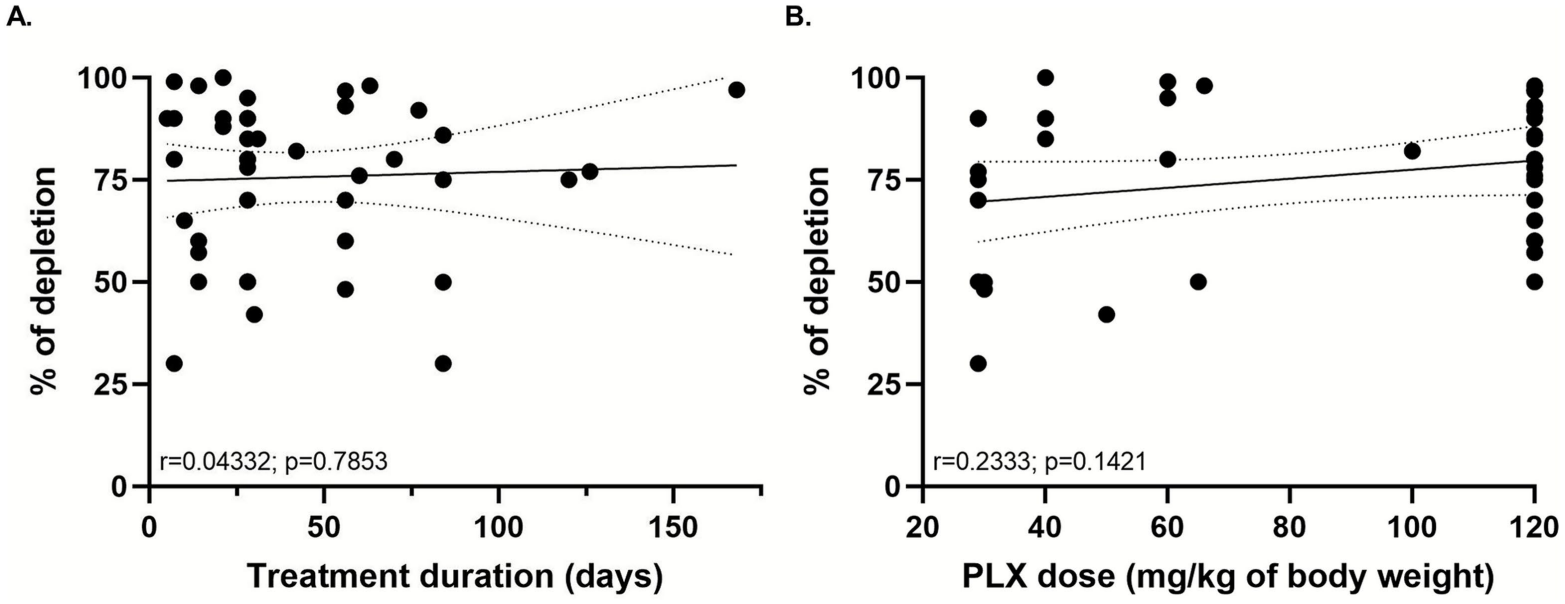
Correlation analysis between the percentage of microglial depletion and treatment duration **(A)** and between percentage of microglial depletion and PLX dose (mg/kg of body weight) **(B)**. Correlation coefficients (Pearson’s r) and associated *p*-values are shown in each panel. Linear regression lines with 95% confidence intervals are illustrated.

It is important to mention that almost all studies reported the validation of microglial depletion protocol by either immunohistochemistry or Western blot for the ionized calcium binding adaptor molecule 1 (Iba-1), a well-known marker of microglial cells. Few studies performed a flow cytometry assay to differentiate microglia from other immune cells (CD11b^+^CD45^low^). Six studies did not report the percentage of microglial depletion induced by their treatment (Dwyer et al., 2020; Oh et al., 2020; Zhang et al., 2021; Guo et al., 2022; Tsai et al., 2020; Son et al., 2023), instead citing other work by the group or others to justify their treatment choice. It is important to note that this represents a potential confound and limits the interpretability of the review. However, as these studies constitute only 14% of the selected studies, we believe that important conclusions can still be drawn. In general, microglial depletion degree diverged among brain regions assessed and duration of PLX-treatment. For instance, there was a discrepancy between studies that assessed the percentage of microglial reduction after 7 days of PLX-treatment. Around 30% of microglia counts was reported after 7 days of treatment (Li et al., 2021), whereas others reported a higher depletion rate with the same period of administration (Liang et al., 2023; Ma et al., 2024; Spangenberg et al., 2016; Shi et al., 2019). The vast majority reported around 50–70%, with some studies showing that the PLX-treatment was able to achieve more than 98% (Spangenberg et al., 2019; Spangenberg et al., 2016; Dodiya et al., 2021; Weigel et al., 2023) or to completely depleted microglia (Shi et al., 2019). Aiming to better understand the possible relationship between (1) percentage of microglial depletion and treatment duration and (2) percentage of microglial depletion and PLX doses employed we performed a correlation analysis (Figure 3). To allow comparison between studies using gavage and those using rodent chow, the daily ingested dose per mouse was estimated based on reported food intake, assuming an average consumption of 0.1 g of chow per gram of body weight, as described by one of the included studies (Johnson et al., 2023). The analysis revealed no significant correlation in either comparison, highlighting the variability in depletion rates across studies and the absence of a consistent pattern.

#### 3.3.3 Main outcomes

The outcomes were divided into two groups: behavior assessment and molecular analysis. In PD, ten studies (62.50%) reported that microglial depletion resulted in neuroprotection (Oh et al., 2020; Jing et al., 2021; Zhang et al., 2021; Guo et al., 2022; Ruan et al., 2022; Liang et al., 2023; Ma et al., 2024; Thi Lai et al., 2024; Iba et al., 2025; Zhang et al., 2025), three studies observed an increase in dopaminergic neuronal death or *α*-synuclein accumulation after PLX-treatment (18.75%) (Yang et al., 2018; Pereira et al., 2023; Stoll et al., 2024), and three studies reported no effect (18.75%) (Dwyer et al., 2020; Li et al., 2021; Abdel-Haq et al., 2022). In addition, the two studies that involved repopulated microglia showed positive outcomes (Li et al., 2021; Bhatia et al., 2023). In AD, five studies (14.71%) reported that microglial depletion resulted in neuroprotection (Spangenberg et al., 2016; Shi et al., 2019; Lodder et al., 2021; Wendt et al., 2022; Johnson et al., 2023), five (14.71%) observed reduction in plaque pathology (Spangenberg et al., 2019; Sosna et al., 2018; Son et al., 2020; Delizannis et al., 2021; Dodiya et al., 2021), seven (20.59%) observed reduction in tau pathology (Asai et al., 2015; Shi et al., 2019; Delizannis et al., 2021; Clayton et al., 2021; Lodder et al., 2021; Karaahmet et al., 2022; Johnson et al., 2023), and two (5.88%) reported reduction in neuroinflammation (Son et al., 2023; Kodali et al., 2024). One study (2.94%) reported worsening synaptic function (Benitez et al., 2021), and other reported increased plaque burden (Clayton et al., 2021). Six studies (17.65%) observed no effects of depletion in plaque load (Dagher et al., 2015; Spangenberg et al., 2016; Unger et al., 2018; Lodder et al., 2021; Wendt et al., 2022; Kodali et al., 2024) and one (2.94%) reported no effect in tau pathology (Bennett et al., 2018). Furthermore, five studies (17.65%) reported data after repopulation of microglia (Spangenberg et al., 2019; Casali et al., 2020; Tsai et al., 2020; Karaahmet et al., 2022; Wang et al., 2023), and observed restoration (Spangenberg et al., 2019), no effect(Karaahmet et al., 2022; Wang et al., 2023), reduced tau pathology (Karaahmet et al., 2022), improvement of cognition, reduced synaptic impairments, and increased neurotrophic factors (Wang et al., 2023), or alterations in plaque morphology from compact to diffuse-like plaque (Casali et al., 2020).

Behavioral outcomes of PD studies were assessed by twelve studies (70.59%) (Yang et al., 2018; Dwyer et al., 2020; Iba et al., 2025; Zhang et al., 2025; Oh et al., 2020; Li et al., 2021; Zhang et al., 2021; Abdel-Haq et al., 2022; Ruan et al., 2022; Bhatia et al., 2023; Pereira et al., 2023; Thi Lai et al., 2024). Five reported an improvement in motor behavior after microglial depletion (41.67%) (Dwyer et al., 2020; Oh et al., 2020; Ruan et al., 2022; Thi Lai et al., 2024; Zhang et al., 2025), two showed worse motor behavior on PLX-treated PD animals when compared to the PD-vehicle groups (16.67%) (Yang et al., 2018; Pereira et al., 2023), two reported no effect on motor behavior after microglia elimination (16.67%) (Abdel-Haq et al., 2022; Iba et al., 2025), one showed improved motor outcome after microglia repopulation (8.33%) (Li et al., 2021), and one reported that microglia replenishment improved cognition assessed by the Y-maze test (8.33%) (Bhatia et al., 2023). Behavioral outcomes of AD studies were assessed by nine studies (34.61%) (Spangenberg et al., 2019; Dagher et al., 2015; Spangenberg et al., 2016; Sosna et al., 2018; Unger et al., 2018; Karaahmet et al., 2022; Weigel et al., 2023; Johnson et al., 2023; Wang et al., 2023). Three studies (33.33%) reported improvement in cognition after depletion of microglia, using tests like contextual fear conditioning, Morris water maze, and novel place recognition task (Dagher et al., 2015; Spangenberg et al., 2016; Sosna et al., 2018). One study (11.11%) reported decrease in anxiety-like behavior using elevated plus maze task (Spangenberg et al., 2019). No studies reported worse effects in cognition after microglial depletion; however, six studies (66.66%) reported no effect using novel object recognition tasks, Morris water maze, contextual fear conditioning, T-maze, and Y-maze tests (Spangenberg et al., 2019; Dagher et al., 2015; Sosna et al., 2018; Unger et al., 2018; Karaahmet et al., 2022; Wang et al., 2023). Two studies (22.22%) reported no effect on motility using open field with depletion (Spangenberg et al., 2019) and repopulation (Karaahmet et al., 2022). Moreover, one study reported improvements in cognition after microglia repopulation, using tests like Morris water maze, contextual fear conditioning, and T-maze (Wang et al., 2023) (the main outcomes extracted from the PD and AD selected studies are described in Supplementary Table 1).

### 3.4 Meta-analysis of the included preclinical studies

#### 3.4.1 Parkinson’s disease studies: dopaminergic neurons

**Figure 4:**
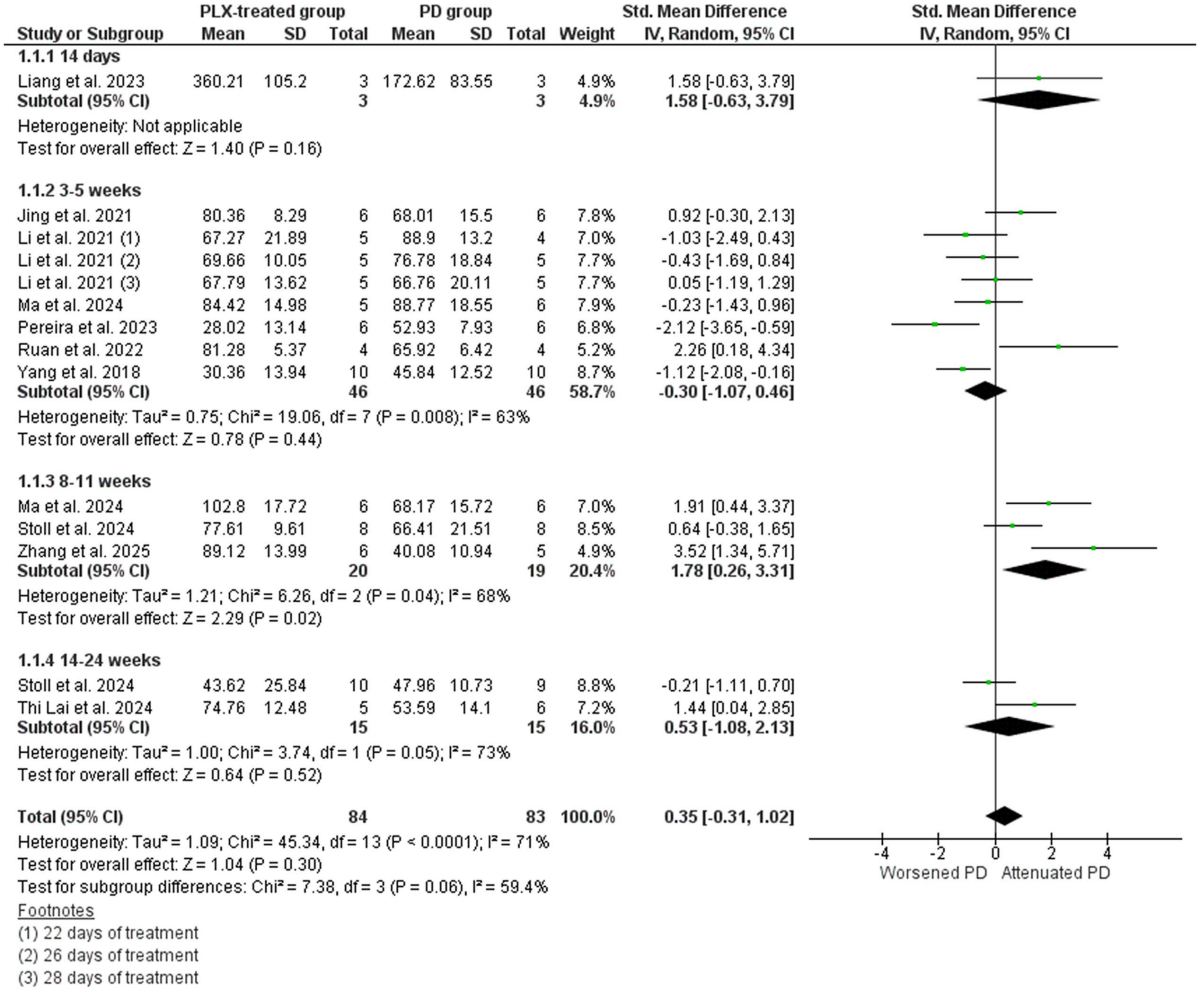
Tyrosine hydroxylase cell counts in the PD vehicle-treated (PD group) and PD PLX-treated (PLX group). Forest plot compares the standardized mean difference (SMD) for the assessed outcome. A random-effect model was applied to meta-analysis. The data markers represent the weight of each study, whereas the diamond shows the overall estimated effect. CI, confidence interval; PD, Parkinson’s disease; SD, standard deviation.

Ten studies that assessed dopaminergic neuronal loss by means of TH-positive cell counts were included in the meta-analysis (Figure 4) (Yang et al., 2018; Jing et al., 2021; Li et al., 2021; Ruan et al., 2022; Liang et al., 2023; Pereira et al., 2023; Ma et al., 2024; Stoll et al., 2024; Thi Lai et al., 2024; Zhang et al., 2025). With the exception of one study that evaluated the locus coeruleus (Jing et al., 2021), all other investigated the SN. The overall data exhibited a marginally increase in TH% in microglia depleted groups; however, polled SMD estimate showed no statistical differences in favor of the PLX-treated group (SMD = 0.35, 95% CI: −0.31 – 1.02; *p* = 0.30) and high heterogeneity (I^2^ = 71%, *p* < 0.0001). The subgroup meta-analysis showed differences only in the 8–11 weeks treated group (*p* = 0.02), suggesting that longer depletion periods might be needed to observe neuroprotection.

#### 3.4.2 Parkinson’s disease studies: *α*-synuclein

**Figure 5:**
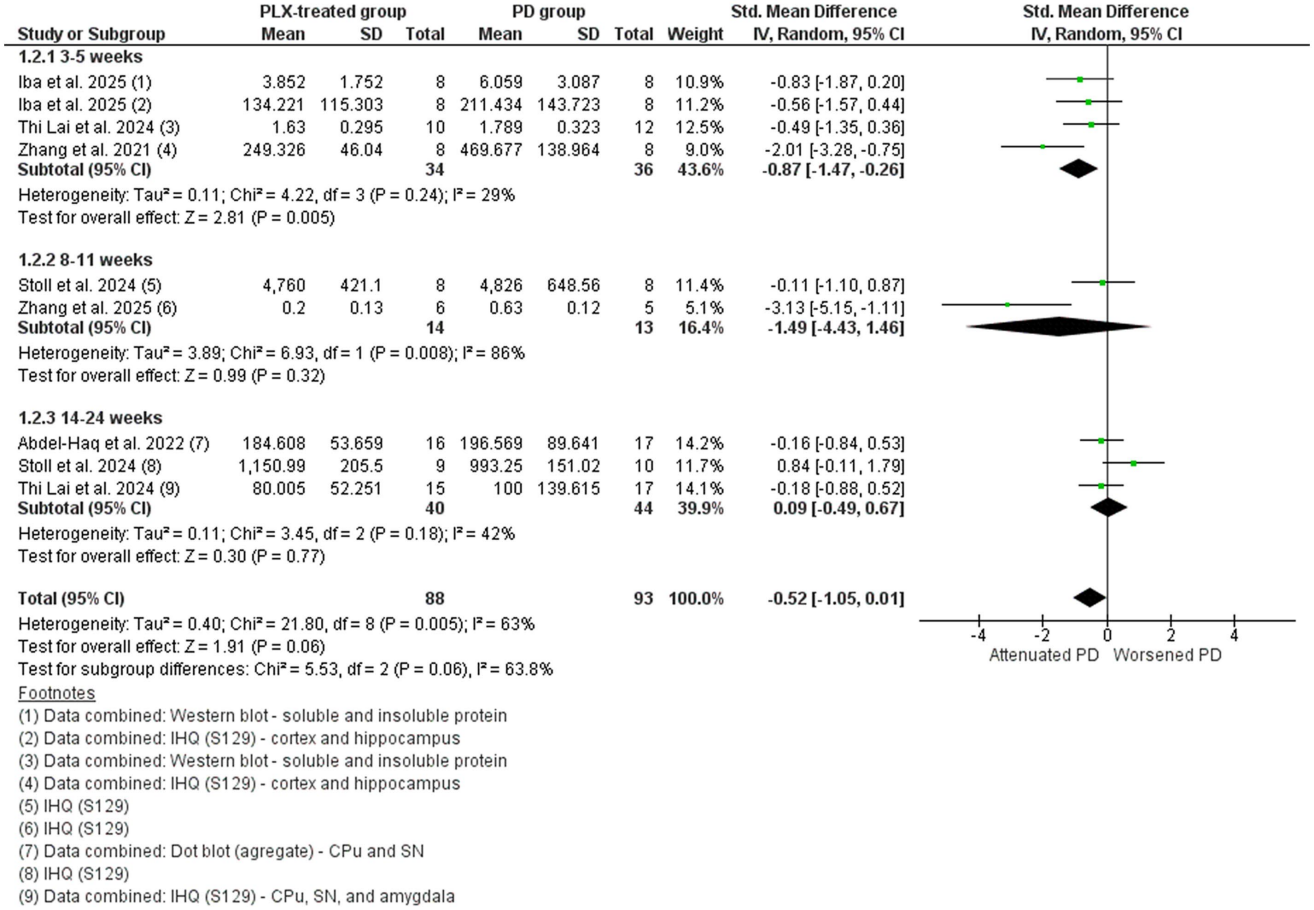
The α-synuclein levels in the PD vehicle-treated (PD group) and PD PLX-treated (PLX group). Forest plot compares the standardized mean difference (SMD) for the assessed outcome. A random-effect model was applied to meta-analysis. The data markers represent the weight of each study, whereas the diamond shows the overall estimated effect. CI, confidence interval; CPu, striatum; IHQ, immunohistochemistry; PD, Parkinson’s disease; SD, standard deviation; SN, substantia nigra.

Six studies that assessed the α-synuclein levels in different brain regions by immunohistochemistry staining of phospho-S129 or Western/dot blot of α-synuclein were included in the meta-analysis (Figure 5) (Zhang et al., 2021; Abdel-Haq et al., 2022; Stoll et al., 2024; Thi Lai et al., 2024; Iba et al., 2025; Zhang et al., 2025). The overall data indicated that the accumulation of α-synuclein in the PD models were reduced by microglial depletion, with polled SMD estimates showing statistically positive tendency in favor of the PLX-treated group (SMD = −0.52, 95% CI: −1.05 – 0.01; *p* = 0.06) and high heterogeneity (I^2^ = 63%, *p* = 0.005). The subgroup meta-analysis showed differences only in the 3–5 weeks treated group (*p* = 0.005), suggesting that shorter depletion periods might impact α-synuclein accumulation in PD.

#### 3.4.3 Parkinson’s disease studies: motor behavior

**Figure 6:**
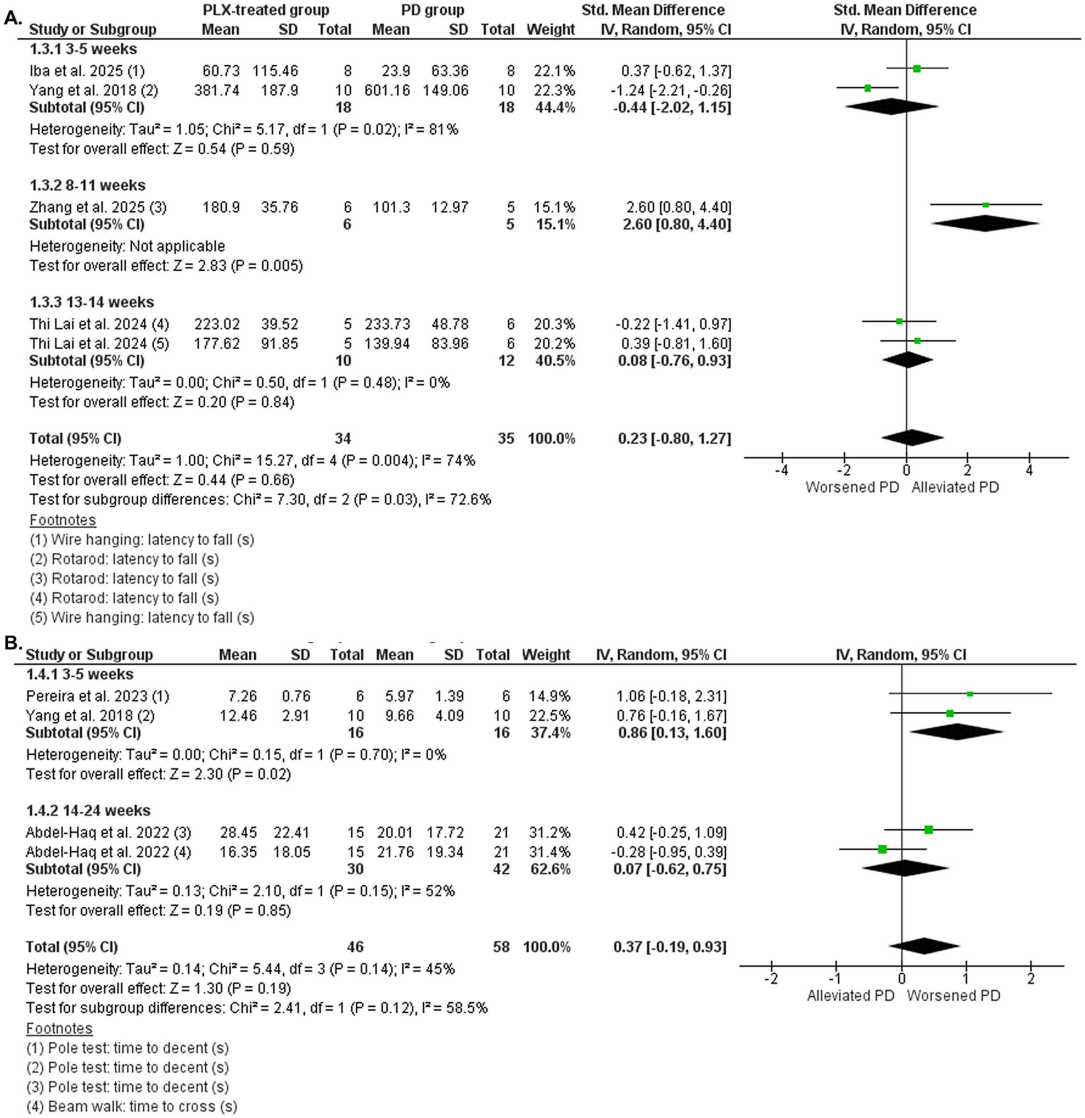
The motor behavior in the PD vehicle-treated (PD group) and PD PLX-treated (PLX group). Forest plot compares the standardized mean difference (SMD) for the rotarod and wire hanging tests **(A)** and pole test and beam walk tests **(B)**. A random-effect model was applied to meta-analysis. The data markers represent the weight of each study, whereas the diamond shows the overall estimated effect. CI, confidence interval; PD, Parkinson’s disease; s, seconds; SD, standard deviation.

Six studies that assessed motor behavior by pole test, beam walk test, wire hanging test, and clasping test were included in the meta-analysis (Yang et al., 2018; Abdel-Haq et al., 2022; Pereira et al., 2023; Thi Lai et al., 2024; Iba et al., 2025; Zhang et al., 2025). Two groups were formed for the meta-analysis: (1) results from rotarod and wire hanging, in which an increase in time indicates a behavior improvement (Figure 6A); and (2) results from pole test and beam walk test, in which a decrease in time indicates a behavior improvement (Figure 6B). The overall data indicated that the performance on rotarod and wire hanging test were not altered by microglial depletion, with polled SMD estimates showing no statistical difference in favor of the PLX-treated group (SMD = 0.23, 95% CI: −0.80 – 1.27; *p* = 0.66) and high heterogeneity (I^2^ = 74%, *p* = 0.004). The subgroup meta-analysis showed a difference only in the 8–11 weeks treated group (*p* = 0.005), which included only one study that showed that microglial depletion improved motor symptoms. In addition, the PLX-treatment had no overall effect on the performance of pole test and beam walk test (SMD = 0.37, 95% CI: −0.19 – 0.93; *p* = 0.19) and medium heterogeneity (I^2^ = 45%, *p* = 0.14). The subgroup meta-analysis showed differences only in the 3–5 weeks treated group (*p* = 0.02), favoring the PD group and suggesting that microglial depletion had detrimental effects on motor behavior. These latter data highlight a worsening of motor dysfunction and given that improvement of motor symptoms is a primary clinical priority, caution needs to be taken when targeting microglia as a therapeutic strategy. However, timing and extent of microglial depletion may critically influence motor outcomes, and these aspects need to be more explored in future studies.

#### 3.4.4 Alzheimer’s disease studies: Aβ

**Figure 7:**
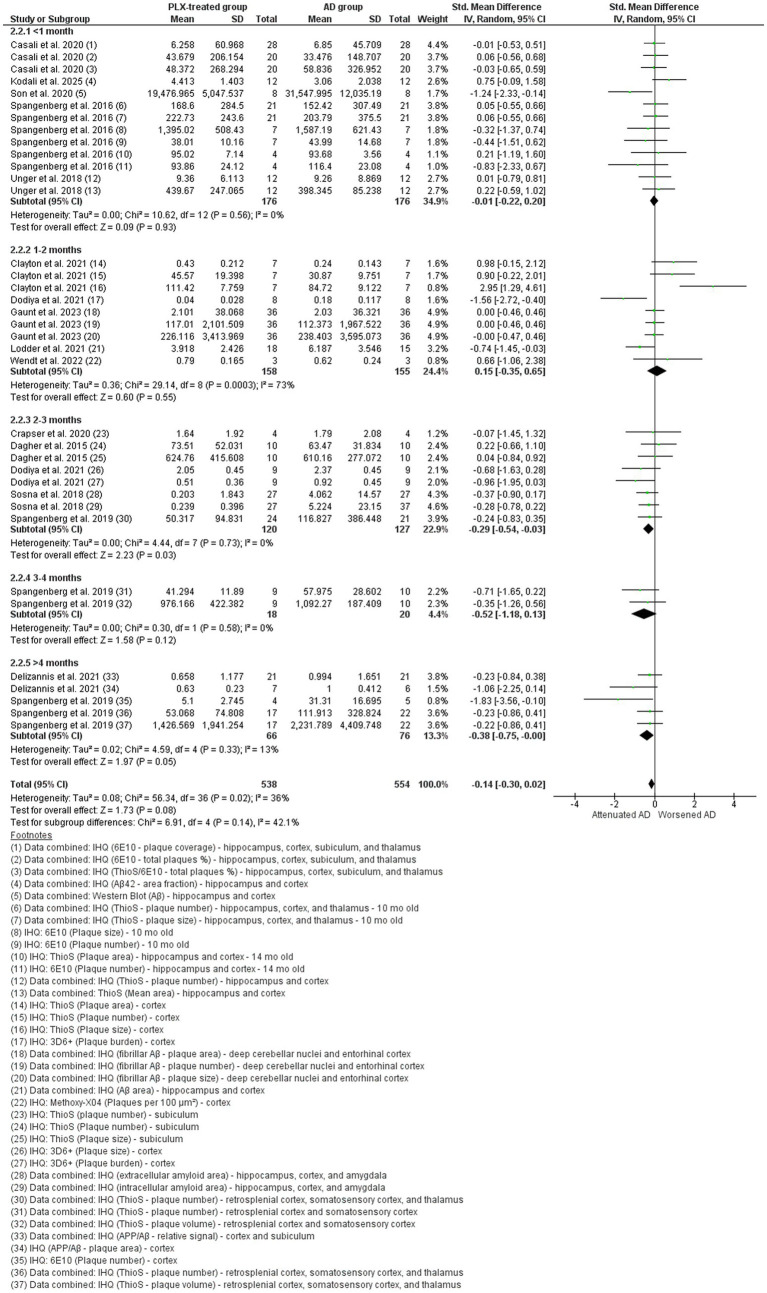
The amyloid-beta (Aβ) levels in the AD vehicle-treated (AD group) and AD PLX-treated (PLX group) after microglial depletion paradigm. Forest plot compares the standardized mean difference (SMD) for the assessed outcome. A random-effect model was applied to meta-analysis. The data markers represent the weight of each study, whereas the diamond shows the overall estimated effect. 3D6^+^: N-terminus of amyloid-beta (Aβ); 6E10: 1–16 amino acid residues of amyloid beta protein; AD, Alzheimer’s disease; APP, amyloid beta precursor protein; Aβ, amyloid beta; CI, confidence interval; IHQ, immunohistochemistry; mo, months; SD, standard deviation; ThioS, Thioflavin S.

**Figure 8:**
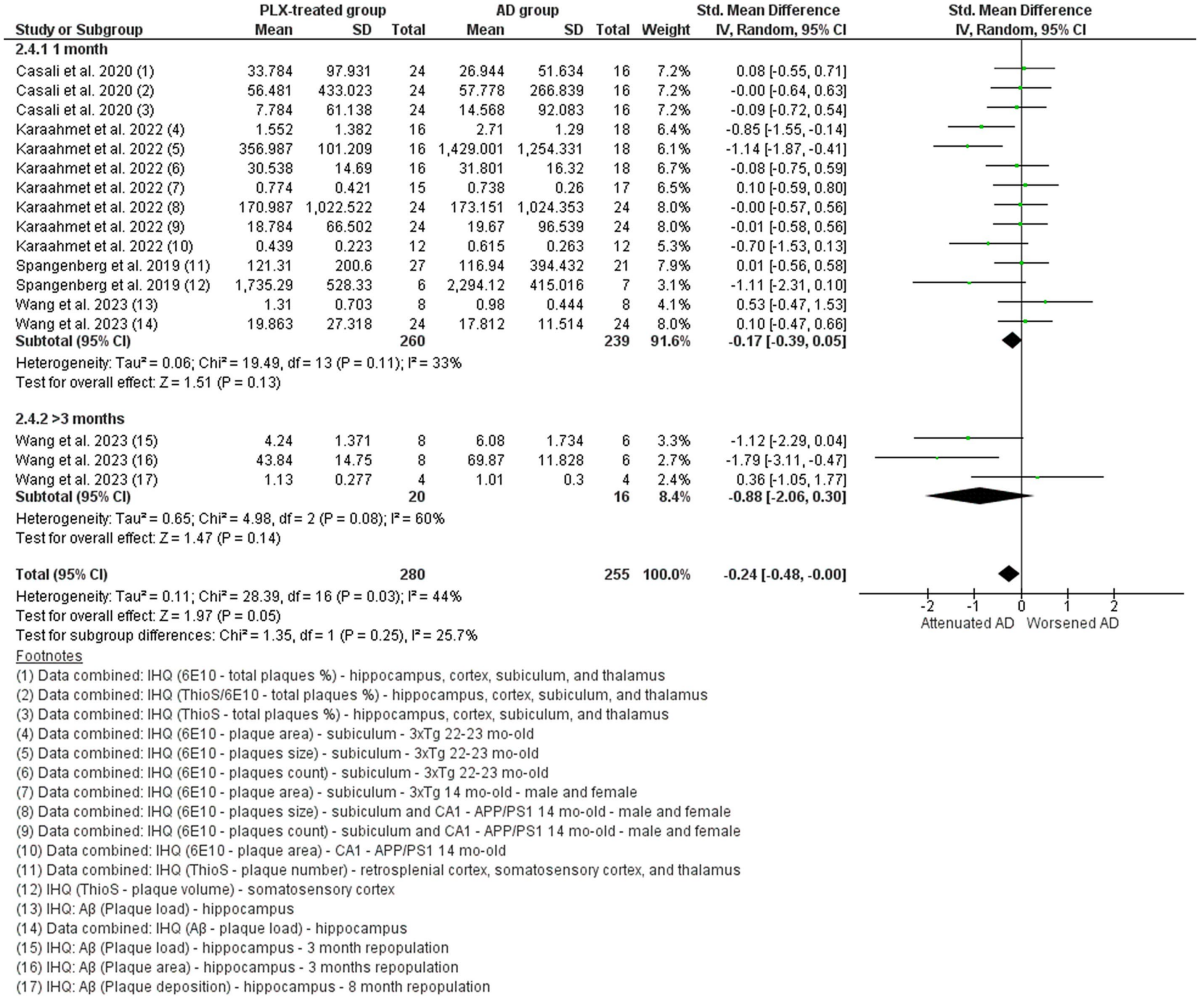
The amyloid-beta (Aβ) levels in the AD vehicle-treated (AD group) and AD PLX-treated (PLX group) after microglial repopulation paradigm. Forest plot compares the standardized mean difference (SMD) for the assessed outcome. A random-effect model was applied to meta-analysis. The data markers represent the weight of each study, whereas the diamond shows the overall estimated effect.6E10: 1–16 amino acid residues of amyloid beta protein; AD, Alzheimer’s disease; APP, amyloid beta precursor protein; Aβ, amyloid beta; CI, confidence interval; IHQ, immunohistochemistry; mo, months; SD, standard deviation; ThioS, Thioflavin S.

Seventeen studies assessed Aβ (Figures 7 and 8) (Spangenberg et al., 2019; Dagher et al., 2015; Clayton et al., 2021; Lodder et al., 2021; Karaahmet et al., 2022; Wendt et al., 2022; Gaunt et al., 2023; Wang et al., 2023; Kodali et al., 2024; Spangenberg et al., 2016; Sosna et al., 2018; Unger et al., 2018; Casali et al., 2020; Crapser et al., 2020; Son et al., 2020; Delizannis et al., 2021; Dodiya et al., 2021). There was a large variation of methods used to access the levels of Aβ in the different studies, including immunohistochemistry, Western Blot, and ELISA assays that evaluated different forms of the protein (e.g., soluble, insoluble, plaque, fibril, oligomers). In light of that, we only included in the meta-analysis data from the immunohistochemistry analysis of either number of plaques, area, or volume, assessed through different staining methods, such as Thioflavin S (ThioS), anti-6E10, anti-Aβ, anti-NAB228, among others. Fifteen studies that showed data from microglial depletion (Figure 7) and four studies that showed data from microglia repopulation (Figure 8) were analyzed separately. For microglial depletion, overall data indicated no difference in Aβ accumulation after PLX-treatment (SMD = −0.14, 95% CI: −0.30 – 0.02 *p* = 0.08) and high heterogeneity (I^2^ = 73%, *p* = 0.0003). However, the subgroup meta-analysis showed differences in the longer treatment protocols, in favor of the PLX-treated group in the 2–3 months (*p* = 0.03) and longer than 4 months treatment paradigm (*p* = 0.05), suggesting that longer depletion periods are needed to observe positive effects on Aβ levels. For microglia repopulation, overall data indicated that microglia repopulation can reduce Aβ levels (SMD = −0.24, 95% CI: −0.48 – 0.00 *p* = 0.05) and low heterogeneity (I^2^ = 44%, *p* = 0.03). For this analysis, two subgroups were created: one month and more than one month repopulation, which include data from 3- and 8-month repopulation. No difference was noted in the subgroup analysis. These data suggest that microglia repopulation might represent an interesting approach, despite more studies are needed to confirm this effect.

#### 3.4.5 Alzheimer’s disease studies: p-tau

**Figure 9:**
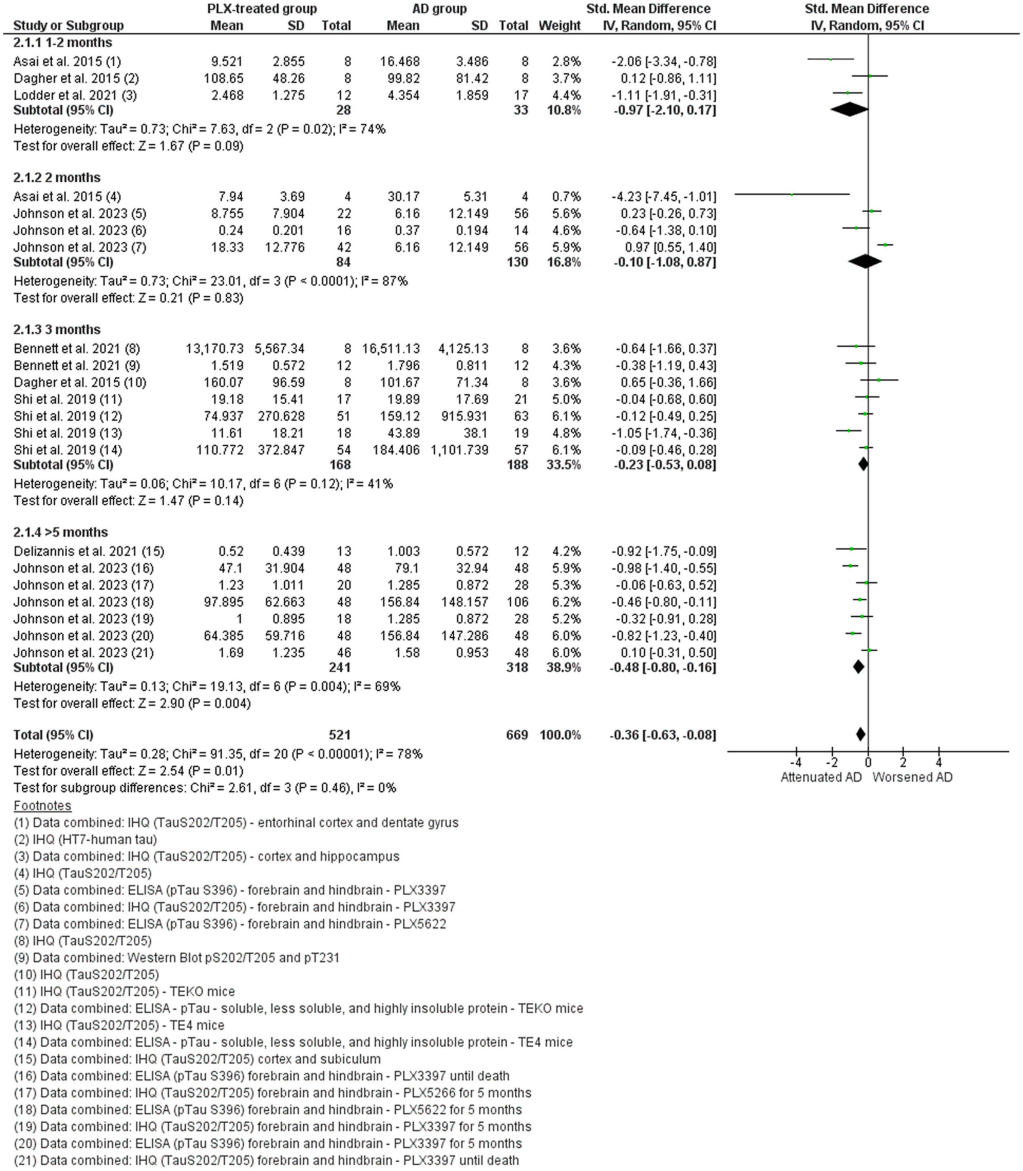
The hyperphosphorylated Tau levels in the AD vehicle-treated (AD group) and AD PLX-treated (PLX group). Forest plot compares the standardized mean difference (SMD) for the assessed outcome. A random-effect model was applied to meta-analysis. The data markers represent the weight of each study, whereas the diamond shows the overall estimated effect. AD, Alzheimer’s disease; CI, confidence interval; ELISA, enzyme-linked immunosorbent assay; IHQ, immunohistochemistry; SD, standard deviation; TE4, ApoE4 knock-in mice; TEKO, ApoE knock-out mice.

Seven studies that evaluated p-Tau levels in different brain regions, including the hippocampus (CA1 and dentate gyrus), entorhinal, somatosensorial, and posterior cortex, subiculum, forebrain, and hindbrain of AD models after PLX treatment were included in the meta-analysis (Dagher et al., 2015; Asai et al., 2015; Shi et al., 2019; Bennett et al., 2018; Delizannis et al., 2021; Lodder et al., 2021; Johnson et al., 2023). The majority of the studies performed immunohistochemistry for p-Tau phosphorylated at S202/T205 (antibody AT8) and/or ELISA assay (Figure 9). The overall data indicated that the accumulation of p-Tau in the AD models were reduced by microglial depletion, with polled SMD estimates showing difference in favor of the PLX-treated group (SMD = −0.36, 95% CI: −0.63 – 0.08; *p* = 0.01) and high heterogeneity (I^2^ = 78%, *p* < 0.0001). In addition, the subgroup meta-analysis showed differences only in the group of longer treatment (more than 5 months) (*p* = 0.004), suggesting that longer depletion periods had larger effects on p-Tau levels.

#### 3.4.6 Alzheimer’s disease studies: cognitive behavior

**Figure 10:**
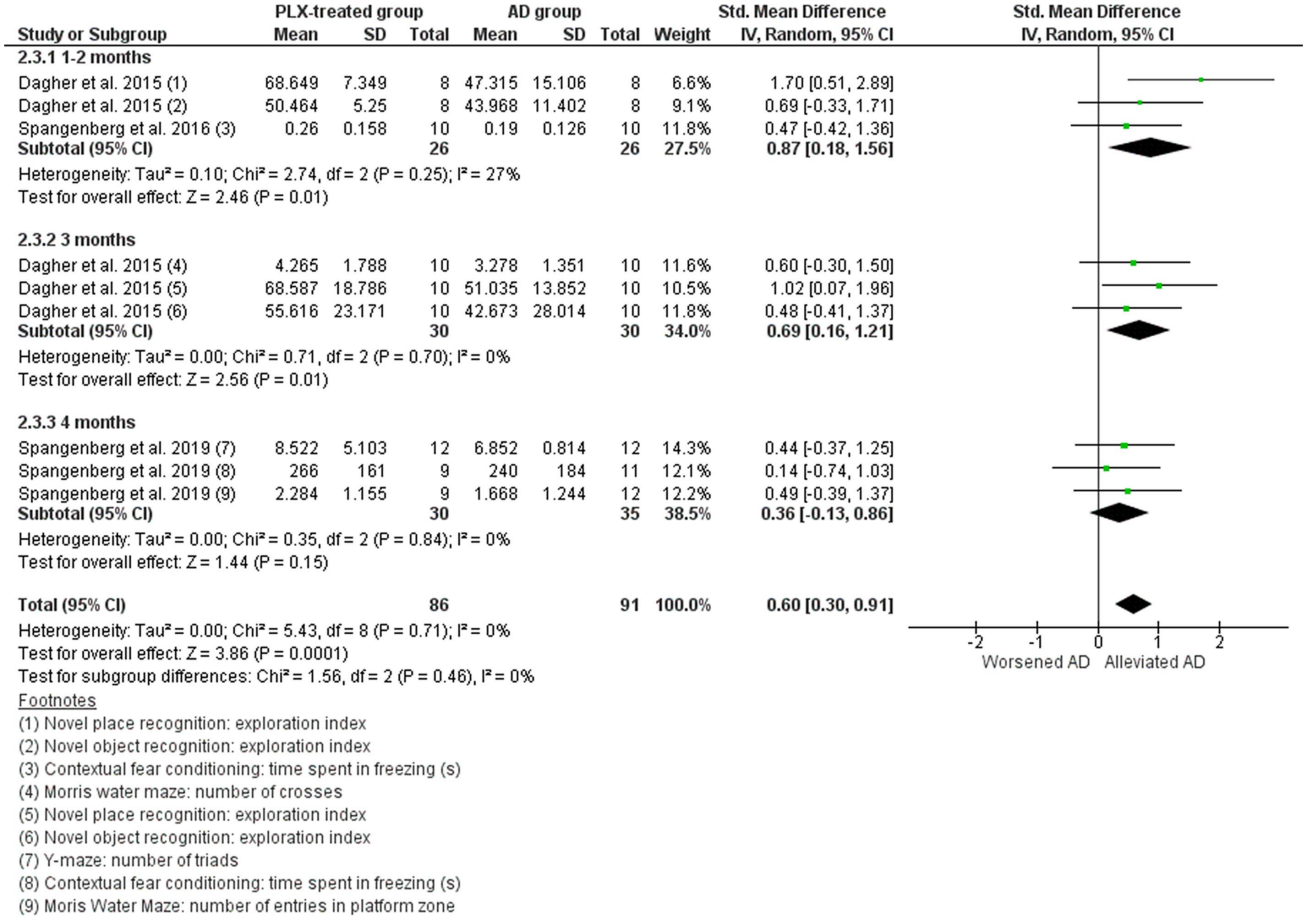
The cognitive behavior in the AD vehicle-treated (AD group) and AD PLX-treated (PLX group). Forest plot compares the standardized mean difference (SMD) for the object recognition, novel place recognition, Y-maze, Morris water maze, and contextual fear conditioning tests. A random-effect model was applied to meta-analysis. The data markers represent the weight of each study, whereas the diamond shows the overall estimated effect. AD, Alzheimer’s disease; CI, confidence interval; s, seconds; SD, standard deviation.

Only three studies were included in the meta-analysis. The other six studies that evaluated behavior were excluded either because they did not assess cognitive behavior or because the relevant data (sample size, mean, or standard deviation) were not available. The three considered studies assessed cognitive behavior by object recognition, novel place recognition, Y-maze, Morris water maze, and contextual fear conditioning tests were included in the meta-analysis (Figure 10), in which an increase in the number, time, or index indicates improved cognitive behavior (Spangenberg et al., 2019; Dagher et al., 2015; Spangenberg et al., 2016). The overall data indicate that the cognitive performance of PLX-treated mice was improved (SMD = 0.60, 95% CI: 0.30–0.91; *p* = 0.0001), and low heterogeneity (I^2^ = 0%). In the subgroup analysis, an effect was observed in the shorter depletion windows: 1–2 months (*p* = 0.01) and 3 months (*p* = 0.01). However, these results need to be carefully interpreted, as data from only three studies were included.

## 4 Discussion

Microglia, the resident immune cells of the central nervous system, play essential roles in maintaining brain homeostasis under physiological conditions. However, in neurodegenerative diseases, these cells can adopt a detrimental phenotype that contributes to disease progression. Accumulating evidence indicates that genetic variants associated with neurodegenerative diseases are preferentially enriched in regulatory elements active in microglia, suggesting that disease risk is largely mediated through alterations in gene regulation rather than coding mutations. Many of these risk variants are linked to immune-related genes highly expressed in microglia (Wickstead, 2023; Yang et al., 2023; Holtman et al., 2024). Given the central role of microglia in immune surveillance and brain homeostasis, dysregulation of microglial gene expression may impair cellular function and contribute to disease onset and progression. Consequently, the depletion of microglia has emerged as a promising strategy and has been investigated in numerous studies. In this review, we compile and analyze the literature on the use of PLX3397 and PLX5622, two well-established CSF1R inhibitors, in AD and PD preclinical models. Overall, we found that microglial depletion appears to exert beneficial effects in both disease contexts. However, these findings are limited by high data heterogeneity and substantial variability in the methodologies employed across studies, with depletion leading to improvements in some pathological pathways while showing no effect, or even adverse effects, in others. The large variability among studies needs to be carefully considered when interpreting the outcomes. In addition, evidence in PD models is limited. Notably, only one study to date has examined the effects of microglial depletion initiated after disease onset, highlighting a significant gap in the literature. Notwithstanding, the majority reported neuroprotective effects after microglial depletion. In AD models, by comparison, a larger number of studies have evaluated depletion at various timepoints, including before, during, and after disease induction. Below, we will discuss in detail what the studies have found so far about the effects of microglial depletion through PLX3397 and PLX5622 treatment in both PD and AD.

### 4.1 Microglial depletion in Parkinson’s disease

Multiple studies have evaluated the effect of microglial depletion using PLX3397 or PLX5622 in mouse models of PD, with many reporting neuroprotective outcomes when depletion occurred prior to or during disease induction (Oh et al., 2020; Jing et al., 2021; Zhang et al., 2021; Ruan et al., 2022; Liang et al., 2023; Ma et al., 2024; Thi Lai et al., 2024; Iba et al., 2025; Zhang et al., 2025). However, a few studies have described detrimental effects, including increased dopaminergic neuron loss and worsened motor impairments following PLX-induced microglial depletion (Yang et al., 2018; Pereira et al., 2023). In these studies, the depletion of microglia was conducted both before and during the disease induction and had a shorter duration (14 days and 30 days). Notably, other studies with comparable intervention timelines reported opposite effects, as the authors reported neuroprotection and positive outcomes. Furthermore, the PD studies included have limitations as only one study has initiated microglial depletion after the onset of pathology (Iba et al., 2025), using the Thy1-*α*-synuclein transgenic model. While neuroprotection was observed in the cortex and hippocampus, the study did not assess behavior or key PD-related outcomes such as motor deficits or dopaminergic neuronal pathology. Additionally, most studies used only male mice and neurotoxin-based models. Only one study included both sexes (Bhatia et al., 2023), and the observed neuroprotective effects were reported only in males. Ages ranged from 5 weeks to 18 months, and treatment durations varied, though a majority used a 21-35-day protocol. The meta-analysis showed no neuroprotection with PLX treatment, despite a slight trend toward increased dopaminergic neurons and benefits in longer treatments (8–11 weeks). Similarly, α-synuclein accumulation showed a trend toward reduced pathology in PLX-treated animals, with shorter treatment durations (3–5 weeks) showing the higher effect. In motor behavior, no improvements were observed after PLX treatment. Interestingly, short-term depletion (3–5 weeks) appeared detrimental to motor outcomes, suggesting that timing and treatment length are critical variables in determining microglial roles in PD progression. Because improving motor symptoms is a primary clinical goal, targeting microglia requires caution, as the timing and extent of their depletion may critically affect motor outcomes. The conflicting results highlight the need for further research to clarify the effects of depletion following disease onset, to explore potential sex differences, and to assess other PD-related pathologies.

### 4.2 Microglial depletion in Alzheimer’s disease

Microglial depletion in AD studies showed great variability; however, most studies still reported beneficial effects, such as neuroprotection (Spangenberg et al., 2016; Wendt et al., 2022; Johnson et al., 2023), reduction of inflammation (Son et al., 2020; Kodali et al., 2024), reduction of Aβ (Spangenberg et al., 2019; Sosna et al., 2018; Dodiya et al., 2021), and of tau pathologies (Asai et al., 2015; Shi et al., 2019; Delizannis et al., 2021; Lodder et al., 2021). Dagher et al. (2015) reported improvements in cognition in mice treated with PLX5622 after disease induction during both 6 weeks and 3 months of treatment. Despite the improvement in cognition, the authors reported there were no effects in Aβ plaque burden. The analysis related to Aβ in the studies of AD were highly variable and this might be related to the methodology used by the authors to access the amyloid plaques data, especially the studies that used 82E1 and 4G8 antibodies. Some studies reported a promising effect of microglial depletion in initial Aβ plaque deposition (Spangenberg et al., 2019; Sosna et al., 2018; Delizannis et al., 2021; Dodiya et al., 2021), whereas other studies reported that microglial depletion was able to reduce neuronal loss without altering plaque load (Spangenberg et al., 2016; Lodder et al., 2021; Wendt et al., 2022). In addition, the age of animals ranged widely, from early postnatal stages to over 21 months, and the majority of studies initiated microglial depletion after pathology was established or during disease progression, with durations ranging from under one month to over five months. The meta-analysis of studies assessing Aβ levels revealed no overall reduction after microglial depletion, although longer depletion protocols (more than 2 months) did show positive effects. PLX treatment demonstrated reductions in p-Tau accumulation, particularly after longer depletion protocols. Cognitive performance showed improvement in PLX-treated mice, suggesting that microglial modulation can positively impact AD-related behavioral outcomes, although more data are needed due to the small number of included studies. These findings suggest that the timing of intervention and the duration of treatment may be key factors in achieving beneficial effects, although the discrepant results point to the need of further studies to corroborate this hypothesis.

### 4.3 The protocols used for microglial depletion

Microglial depletion in PD and AD was achieved by using two CSF1R inhibitors, PLX3397 and PLX5622. These were used in equal proportions across studies, which PLX3397 in 52.17% and PLX5622 in 47.83% of cases. However, a wide variability was observed in: (i) dosage, (ii) treatment duration, and (iii) the timing of intervention (before, during, or after disease onset). The most common route of administration was chow, although some used gavage. PLX5622 was consistently dosed at 1200 mg/kg of chow. PLX3397 dose varied, with some studies using relatively low doses (~300–600 mg/kg), whereas others applied higher doses (~1,000 mg/kg). In AD, two studies, and in PD, six studies, used gavage for PLX administration. The doses generally ranged from 30 to 65 mg/kg. The typical duration of microglial depletion treatment was approximately 30 days. In PD models, most studies employed treatment periods ranging from 21 to 35 days. In contrast, AD studies showed a more balanced distribution, with durations either around one month or between one to two months, whereas some studies extended the treatment to longer periods. Despite this variability, both low and high doses achieved at least 50% of microglial depletion in the majority of studies and brain regions examined. Most studies reported some remaining microglia after treatment with PLX. Apart from Spangenberg et al. (2016, 2019) that reported depletion of 99–100%, most studies reported partial depletion, with efficiency varying according to brain region, strain, dosage, and treatment duration. These findings suggest that microglial depletion via CSF1R inhibition is typically neither complete nor homogeneous, and cannot be attributed solely to PLX drug dosing, but instead could reflect a more complex interplay of experimental and biological factors. In addition, no correlation was observed between the depletion rate and PLX-treatment duration or dose. However, it is important to note that there is a high divergence between studies when we consider the brain region evaluated.

Treatment duration varies across studies. Longer treatment periods are often used to maximize microglial depletion or to assess sustained effects on pathology and behavior, whereas shorter protocols typically aim to evaluate acute microglial contributions during early disease stages. One study demonstrated that longer treatment durations combined with higher doses of PLX3397 resulted in more effective microglial depletion compared to shorter treatments with lower doses, suggesting that PLX3397 acts in a concentration- and time-dependent manner (Wang et al., 2023). However, prolonged microglial depletion may have unintended consequences. Indeed, two studies reported detrimental effects following long-term PLX treatment (>3 months). Dodiya et al. (2021) found that short-term PLX5622 reduced Aβ plaque burden, but no effect was observed with a long-term treatment. Similarly, Stoll et al. (2024) showed that 2 months of PLX3397 treatment did not affect the number of α-synuclein-positive neurons in the SN, but extending treatment to 6 months increased both α-synuclein expression and microglial soma size, suggesting that prolonged PLX3397 treatment may be detrimental. These findings indicate that treatment duration influences both depletion efficiency and disease outcomes, and that prolonged depletion may generate compensatory microglial responses. However, this variability makes cross-study comparisons difficult and underscores the importance of carefully aligning depletion duration with disease stage and experimental goals.

The timing of intervention relative to disease progression also appears to be one of the key factors in determining whether microglial depletion is beneficial. Microglial depletion initiated before and during disease promoted neuroprotection in PD (Oh et al., 2020; Jing et al., 2021; Zhang et al., 2021; Ruan et al., 2022; Liang et al., 2023; Ma et al., 2024; Thi Lai et al., 2024; Iba et al., 2025; Zhang et al., 2025) and AD (Spangenberg et al., 2016; Wendt et al., 2022; Johnson et al., 2023). Furthermore, it was reported a reduction of plaques (Spangenberg et al., 2019; Sosna et al., 2018; Delizannis et al., 2021; Dodiya et al., 2021; Son et al., 2023) and tau pathology (Asai et al., 2015; Shi et al., 2019; Delizannis et al., 2021; Clayton et al., 2021; Karaahmet et al., 2022; Johnson et al., 2023). Although some beneficial effects have been reported when depletion occurred after disease onset, these findings were inconsistent, and at least one study in AD noted a worsening of amyloid pathology. In PD, data on PLX administration after disease induction remains limited. In summary, before disease onset, microglia are not yet in a proinflammatory or disease-associated state. Thus, it seems more rational to deplete microglia after the onset/early stages of disease, when intervention may prevent their detrimental activation.

Most studies in PD evaluated male mice, whereas in AD both males and females were used. Although it is known that those diseases affect men and women differently, the sex must be considered in protocols of microglia depletion. Male mice showed a higher level of microglial reduction than females when treated with the same chow: Shi et al. (2019) used only male mice in their study because female showed decreased percentage of microglial depletion. These data were assessed based on plasma levels of mice treated with PLX3397. Johnson et al. (2023) also discuss the sex-specific effects of PLX: they showed that there was an increase in activation of inflammation-related pathways in PLX-treated male mice compared to female mice, suggesting that therapeutic targets that focus on microglia must consider sex-dependent effects. The sex-specific effects of PLX were also reported in behavioral assessments. Bhatia et al. (2023) observed that males but not females showed an increase in time spent active, increase in maximum speed, in time spent rearing and time spent moving. Altogether, these data suggest that biological sex can modify the impact of PLX5622 in hyperactivity and anxiety-like behaviors. Furthermore, again in males but not in females, they showed decrease in α-synuclein in olfactory peduncle (Bhatia et al., 2023). Wang et al. (2023) also reported that the duration of treatment of PLX3397 resulted in distinct percentages of depletion in males and females. Treatment during 7 and 14 days depleted a higher percentage in males when compared to females, but 21 days resulted in the opposite, suggesting that females may need more time to get microglia depleted when compared to males (Wang et al., 2023).

The parent-of-origin of transgenes is also a crucial factor when discussing drug testing and the mechanisms involved in neurodegenerative diseases such as AD and PD. This variable is particularly important when analyzing divergent effects across mouse models. For example, the 5xFAD mouse, a widely used model of AD, presents a specific feature that may influence both microglial depletion and disease pathology, including Aβ accumulation. Transgenic inheritance modulates transgene expression, and Aβ levels may be higher when the transgene is inherited paternally rather than maternally (Sasmita et al., 2025). Consequently, the extent of microglial depletion and its outcome may differ depending on the parental origin of the transgene. This highlights an important factor to consider when using mouse models, as accounting for parent-of-origin effects can improve data interpretation and enhance study reproducibility. In addition, this might explain the variability observed among studies that used the same model and a similar treatment protocol.

Heterogeneity in PLX-treatment regimens likely reflects differences in experimental duration, treatment time, disease models, and sex. Studies aiming to achieve partial versus extensive microglial depletion, as well as those targeting distinct disease stages, therefore employ different doses and treatment durations. Importantly, the timing of microglial depletion relative to disease progression appears to be a key determinant of the outcome. These discrepancies likely arise from differences in microglial functional states, as microglia transition from homeostatic to disease-associated phenotypes over disease progression (Heneka et al., 2025; Tansey et al., 2022). In addition, biological sex further modulates the effects of PLX treatment, with several studies reporting sex-dependent differences in depletion efficiency, inflammatory pathway activation, and behavioral outcomes under identical dosing regimens (Bhatia et al., 2023; Wang et al., 2023). Together, these factors indicate that variability in PLX treatment cannot be interpreted in isolation but rather reflects complex interactions between drug exposure, disease stage, brain region, and sex. This complexity likely contributes to divergent experimental outcomes and limits cross-study comparability, highlighting the need for more standardized studies in future work.

### 4.4 Advantages of using PLX3397 or PLX5622 as microglial inhibitors

PLX3397, PLX5622, and other microglial inhibitors primarily act by blocking the CSF1R, which is critical for microglial survival and proliferation (Spangenberg et al., 2019). Other available inhibitors, such as Ki20227, JNJ-40346527, and GW2580, also target CSF1R and can reduce inflammation. However, their primary mechanism appears to be the inhibition of microglial proliferation rather than actual depletion of microglia from the central nervous system. For instance, GW2580 treatment did not alter baseline microglial levels, suggesting it limits activation and expansion without eliminating resident microglia (Neal et al., 2020; Gerber et al., 2018). INJ-40346527 was reported to block microglia proliferation and inflammation, alleviating the pathology progression in a mouse model of AD (Mancuso et al., 2019); but it is mostly an anti-proliferative agent rather than a full depletion agent under moderate doses. In addition, these inhibitors are not specific to CSF1R. Ki20227 can bind to the vascular endothelial growth factor receptor-2 (KDR/VEGFR-2), stem cell factor receptor (c-Kit), and platelet-derived growth factor receptor beta (PDGFRβ) (Ohno et al., 2006). Notwithstanding, PLX3397 and PLX5622 can in fact eliminate microglia from the brain and alter the phenotype of remaining microglia. Elmore et al. (2014) reported that PLX-treatment induces microglial death, as microglia were positive for active caspase-3, a marker of apoptosis, and propidium iodide, indicative of dying cells.

Microglial depletion through CSF1R inhibition may represent a more selective approach, as it targets microglia without broadly affecting other immune cells. In line with this, Okojie et al. (2023) assessed the peripheral immune profile in lymphoid (spleen and bone marrow) and non-lymphoid (lungs, kidney, and heart) organs. They reported that microglial depletion with PLX3397 (660 mg/kg for 7 days) did not significantly alter peripheral immune cell populations in either lymphoid or non-lymphoid tissues, except for the heart, where a minimal reduction was observed in CD3 + cells, inflammatory and patrolling monocytes, and CD11b + Ly6G + neutrophils. The most pronounced reduction occurred in the CNS, where decreases were observed in CD45 + macrophages, CX3CR1GFP/+ cells, and CD11b + CD45-intermediate microglia (Okojie et al., 2023). Johnson et al. (2023) further evaluated PLX73086, a CSF1R inhibitor structurally related to PLX3397 and PLX5622 but lacking brain penetrance. Their aim was to determine whether blocking peripheral cells via CSF1R inhibition could influence tau pathology in the brain. Chronic treatment with PLX73086 had no effect on microglial numbers, as assessed by Iba-1 and P2yr12, nor on tau levels. These findings suggest that PLX3397 and PLX5622 are more effective in modulating CNS pathology, likely due to their ability to cross the blood–brain barrier (Johnson et al., 2023). Central routes of administration, such as intraventricular, intrathecal, or intracerebral delivery, could further improve specificity and could allow future studies to more precisely distinguish between central versus peripheral contributions of microglia in the neurodegenerative disease context. Although we cannot rule out the possibility that the effects observed following PLX treatment are not solely attributable to microglial elimination, as other immune cell populations may also be affected, microglia are consistently shown to be depleted, indicating that the observed effects largely reflect microglial loss. Nonetheless, the development of more specific inhibitors is needed to confirm this hypothesis.

### 4.5 Microglia repopulation

Unlike neurons, microglia have the capacity to repopulate. Following depletion, they can quickly proliferate through mitosis, allowing rapid repopulation within a short period. The brain can be fully repopulated with microglia within 5–7 days after cessation of CSF1R inhibition (Wang et al., 2023; Huang et al., 2018). The selected studies in both AD and PD models have reported interesting effects, including cognitive improvements following microglial repopulation. Li et al. (2021) observed a very interesting effect of repopulation in PD mice model. When the disease was induced on the day the PLX treatment stopped there was no effect of repopulation; however, when PD was induced after 7 days of withdrawal, an increase in dopaminergic neurons in SNc was reported (Li et al., 2021). Similarly, Bhatia et al. (2023) observed that 7 days of withdrawal after PD induction was neuroprotective in males with decrease of pSer129- *α*-synuclein and pan-α-synuclein, and decreased pSer129- α-synuclein in the olfactory peduncle and the lateral limbic rhinencephalon. Interestingly, those effects were not observed in females, which reinforces the distinction not only of microglial depletion in both sexes as discussed previously but also of repopulation. More studies evaluated repopulation in AD models, and they reported positive effects. Despite the meta-analysis revealed no overall reduction of Aβ levels after one month repopulation, longer repopulation protocols (more than 2 months) did show positive effects, indicating repopulation might be a beneficial approach.

Microglia appear to exhibit distinct characteristics after repopulation. Some studies suggest that repopulated microglia adopt a non-inflammatory phenotype (Kodali et al., 2024), whereas others propose that they may arise from PLX-resistant microglia, which can display an activated profile and pro-inflammatory phenotype (Lodder et al., 2021). Stoll et al. (2024) demonstrated that long-term treatment with PLX3397 enhanced the pro-inflammatory profile of the remaining microglia. Similarly, Iba et al. (2025) reported that the residual microglia showed an increased cell body area, not only in transgenic mice but also in non-transgenic mice treated with PLX. A previous study showed that *csf1r* expression is downregulated in activated microglia in neurodegenerative conditions (Krasemann et al., 2017), suggesting that activated microglia are less dependent on the CSF1R signaling pathway, and therefore may be less susceptible to apoptosis induced by blockade of CSF1R signaling (Shi et al., 2019). Furthermore, plaque-associated microglia express lower levels of the *csf1r* gene compared to distal microglia in AD mice models (Le et al., 2024). Several studies also indicate that some microglia persist despite high PLX doses, suggesting the existence of PLX-resistant populations. In AD mouse models, these resistant cells are often plaque-associated and exhibit a pro-inflammatory phenotype.

Huang et al. (2018) reported that the microglial transcriptome after repopulation differs from that of resident microglia. Although few studies included in this review directly evaluated microglial repopulation, they reported beneficial effects in AD and PD models following repopulation, even when microglial depletion alone did not produce positive outcomes. This suggests that microglial repopulation may act as a cellular “reset,” restoring a more homeostatic morphology and promoting an anti-inflammatory profile. Acute microglial depletion also leads to the infiltration of peripheral monocytes into the central nervous system, and notably, these cells exhibit a behavior similar to that of resident microglia. In addition, acute depletion promotes the proliferation of residual microglia rather than *de novo* differentiation from microglial progenitors (Yoo and Kwon, 2021). This mechanism may help to explain the observed differences between microglial transcriptomes before and after repopulation.

In summary, complete microglial depletion may not be a viable translational strategy, as its effectiveness likely depends on disease stage, dose, and treatment duration. However, periodic depletion through short-term CSF1R inhibition followed by repopulation could represent a more promising approach (Wang et al., 2023; Kodali et al., 2024).

## 5 Limitations and future perspectives

This systematic review has several limitations that should be considered when interpreting the findings. First, all included studies were preclinical, highlighting the need for well-designed clinical studies to determine the translational relevance of microglial depletion strategies. Future translational efforts may need to focus on transient, partial, or state-specific modulation of microglial activity, improved CNS selectivity, or targeting downstream inflammatory pathways, rather than broad CSF1R blockade. Such approaches may offer a more feasible option when we consider the translational aspect, particularly in vulnerable elderly populations that constitute the majority of AD and PD patients. Notably, PLX3397 is already approved for clinical use as TURALIO® in a different disease context and has been demonstrated continued benefit in patients with tenosynovial giant cell tumor, including long-term symptom relief in longitudinal studies (Dai et al., 2025). This clinical experience supports that PLX is safe and feasible in humans, establishing a potential use for other diseases where microglia play a central role in pathophysiology, such as neurological disorders, which needs to be better explored in future studies to eliminate possible risks for those vulnerable populations.

Additionally, substantial heterogeneity was observed across studies with respect to animal models, treatment duration, dosage, timing of intervention, and outcome assessment methods. This variability likely contributes to the limited or inconsistent statistical effects observed in some of the meta-analyses. Indeed, meta-analytical heterogeneity was extremely high for several outcomes, and risk of bias was generally high across the included studies. Although pooled analyses suggest potential effects, the high heterogeneity indicates that these findings must be interpreted with extreme caution and cannot be considered definitive evidence of therapeutic efficacy. These limitations underscore the need for more standardized, rigorous, and high-quality preclinical studies. Furthermore, only three studies contributed data to the cognitive and behavioral meta-analysis in AD models, limiting the robustness of these conclusions. This is particularly noteworthy, given that AD models were more frequently used overall (*n* = 26), yet only nine studies assessed behavioral outcomes in those models. In contrast, although fewer studies employed PD models (*n* = 17), more than half (*n* = 12) included behavioral assessments. Finally, in PD models, the timing of microglial depletion was relatively consistent across studies, with interventions typically initiated before or during disease induction. While this approach facilitates a mechanistic investigation, it may not accurately reflect clinical therapeutic scenarios. Few studies have examined the effects of PLX treatment after disease onset, representing an important gap that warrants further investigation.

Another limitation that needs to be stressed out is the off-target effects of PLX3397 and PLX5622. Although effective in the central nervous system, there might be effects on peripheral immune cells, raising concerns about their specificity and translational potential (Claeys et al., 2023). Additionally, the duration and regimen of PLX administration differ widely across studies, making it difficult to establish optimal depletion protocols. Future research should explore partial and intermittent depletion, or either repopulation strategies to better model clinical scenarios and reduce potential systemic effects. Moreover, the methodological quality of included preclinical studies was generally low, with frequent omissions in blinding, randomization, and reporting unclear methods, further limiting the interpretation of results and the strength of conclusions. Many PD studies only used male mice, restricting the generalizability of findings across sexes. Finally, a critical limitation in AD research is the incomplete pathology represented by some mouse models, such as 5xFAD, which do not develop tau pathology, or Tau-based models, such as Tg2541 and Tg4510, which do not include amyloid pathology. This weakens the translational validity of findings.

Collectively, these limitations highlight the need for more rigorous preclinical studies and clinical interventions to better understand the role of microglia in AD and PD and the possible beneficial effect of its elimination in a disease context. The limitations discussed above further suggest that alternative microglial modulation strategies may constitute promising directions for future investigation, as they could represent more refined therapeutic advantages compared to broad microglial depletion. Those strategies may include, for example, microglial preconditioning, phenotypic reprogramming, selective pathway inhibition, and the generation of more robust evidence regarding depletion followed by repopulation. In addition, a deeper understanding of how genetic variation within the immune system influences neurodegenerative diseases is clearly desirable.

## 6 Conclusion

In conclusion, this systematic review and meta-analysis highlight microglial depletion as a promising approach for modulating disease-related pathology in Alzheimer’s disease (AD) and Parkinson’s disease (PD), despite an obvious need for cautious interpretation of its therapeutic potential. Our findings also underscore key gaps in the current literature that warrant further investigation. These include the need for higher inclusion of both sexes, particularly in PD models, more behavioral assessments in AD models, and inclusion of more intervention periods, especially in post-disease onset treatments in PD. Additionally, alternative approaches, such as partial or intermittent microglial depletion, repopulation, microglial preconditioning, phenotypic reprogramming, selective pathway inhibition, and genetic variations remain poorly understood and should be further explored. Overall, this review may serve as a valuable resource for guiding future research aimed at clarifying the complex role of microglia in neurodegeneration and refining microglia-targeted potential therapeutic strategies for AD and PD.

## Data Availability

The original contributions presented in the study are included in the article/Supplementary material, further inquiries can be directed to the corresponding author.
