# Supplementary material for: The impact of CSF1R inhibitor-mediated microglial depletion in rodent models of Alzheimer’s and Parkinson’s disease: a systematic review and meta-analysis
Source: Front Aging Neurosci. 2026 Feb 25;18:1733682. doi: 10.3389/fnagi.2026.1733682 (PMC12975910; doi:10.3389/fnagi.2026.1733682)
Supplement: Supplementary file 1 [file Supplementary_file_1.docx]

**Database search strategies**

**Total n=3,195**

**Pubmed N=665**

(((plx3397[Title/Abstract]) OR (plx5622[Title/Abstract])) OR (CSF1R Inhibitor[Title/Abstract])) OR (Pexidartinib[Title/Abstract])

**Web of Science N=764**

Plx3397 (Abstract) or plx5622 (Abstract) or CSF1R Inhibitor (Abstract) or Pexidartinib (Abstract)

**EMBASE N=1,766**

#1 plx3397:ab,ti

#2 plx5622:ab,ti

#3 pexidartinib:ab,ti

#4 csf1r AND inhibitor:ab,ti

#1 OR #2 OR #3 OR #4
