# Supplementary material for: The impact of CSF1R inhibitor-mediated microglial depletion in rodent models of Alzheimer’s and Parkinson’s disease: a systematic review and meta-analysis
Source: Front Aging Neurosci. 2026 Feb 25;18:1733682. doi: 10.3389/fnagi.2026.1733682 (PMC12975910; doi:10.3389/fnagi.2026.1733682)
Supplement: Supplementary file 2 [file Table_1.docx]

**Supplementary Table 1:** Main outcomes of the selected studies.

|  | | **Behavioral results** | **Molecular results** | **Summary outcome** |
| --- | --- | --- | --- | --- |
| *Parkinson’s disease* | | | | |
| Yang et al. | 2018 | Rotarod: ⇩ latency to fall  Pole test: ⇧ time do descent the pole | IHQ: ⇩TH cell number in the SNc and density in the CPu Flow cytometry: ⇧ infiltration of CD4+ T cells, CD8+ T cells, monocytes, macrophages, and neutrophils; ⇧TNFα, IL-6, iNOS, and chemokines like IP-10 and CCL2 RT-PCR: ⇧IL-1β, TNFα, IL-2, IL-6, IFNγ, and iNOS ELISA: ✖ BDNF levels | Worsened degeneration and motor impairment |
| Dwyer et al. | 2020 | Improved spontaneous home cage locomotor activity | IHQ: ✖ TH counts in the SNc; prevented morphological alterations in Iba-1-positive cells in the SNc; ⇧GFAP ELISA: ✖ IL-6, corticosterone, TNFα, IL-10 in the plasma;  ⇧ soluble α-syn in the CPu WB: ⇧ total alfa-syn in the CPu  ⇩ CX3CR1, caspase 1 and 3 in the SNc  ⇧ Sirtuin3 in the SNc | No effect on degeneration |
| Oh et al. | 2020 | Adhesive Removal Test: ⇩ time to remove the adhesive Forced Swim Test: ⇩ immobility time | PET scan: ⇩ neuroinflammation (TSPO), dopaminergic transporter, and glutamate receptor subtype 5 tracer uptake in the CPu;  WB: ⇩Iba-1 density in the CPu | Neuroprotective |
| Jing et al. | 2021 | NA | IHQ: ⇧TH in locus coeruleus | Neuroprotective |
| Li et al. | 2021 | *Repopulation:* Pole test: ⇩ time to turn around | *Microglia depletion:* IHQ: ⇩TH counts in the SNc 1d after model induction, ✖ after 5 or 7d; ✖ TH density in the CPu  ⇩Iba-1 counts in the SNc and CPu  ✖ GFAP counts in the SNc and CPu WB: ✖ TH, GFAP, TNFα, IL1β, COX2 and iNOS in the CPu, caspase1, IL18 in the SNc; ⇩ NLRP3, apoptosis-associated speck-like protein (ASC), C1q, IL1α, TNFα, IL6 in the SN and C1q, IL1α and IL6 in the CPu ELISA: ⇩ IL1α, TNFα, IL1β, IFNγ, IL6, IL2, IL4, CCL22, IL13 in the SNc; IL2 in the CPu RT-PCR: ⇩ C1q, IL1α, TNFα, IL6; ✖ IFNy, COX2 and iNOS Chromatography: ✖ DA, DOPAC, HVA, 5-HT and 5-HIAA *Microglia repopulation:* Repopulation 1: no effect Repopulation 2: IHQ: ⇧TH, ✖ Iba-1, ⇧GFAP in the SNc  RT-PCR: ⇧Iba-1, GFAP, MHC I, MHC II, BDNF, NGF, TGFβ1, IGF1, TREM2 in the CPu | No effect on degeneration when microglia was depleted, neuroprotection when PD was induced after repopulation |
| Zhang et al. | 2021 | MWM: ⇩ escape latency, traveled distance, and latency for first platform crossing; ⇧ platform crossing number and time spent in target quadrant NORT: ⇧ recognition index Passive avoidance test: no difference | IHQ: ⇧ NeuN counts and PSD95 density  ⇩Ser129-phospho-α-syn in HPC and cortex  normal morphology and ⇩GFAP density TUNEL assay: ⇩TUNEL+ cells WB: ⇧ NeuN and PSD95 in HPC and cortex  ⇩ caspase 3 and Bax and ⇧Bcl-xL in HPC and cortex RT-PCR: ⇩ iNOS, TNFα, and IL-1β in HPC and cortex GSH and MDA assay: ⇩ MDA levels and ⇧GSH in HPC and cortex | Neuroprotective |
| Abdel-Haq et al. | 2022 | Pole test: no effect Beam Walk: no effect | Dot blot: ✖ aggregated αSyn | No effect |
| Guo et al. | 2022 | NA | Evan blue: ⇩ BBB permeability in HPC and cortex WB: ⇧ ZO-1, claudin-5 and occludin in HPC and cortex  ⇩ MMP-2/-9  IHQ: ⇧ ZO-1, claudin-5 and occludin in HPC and cortex | Decreased BBB permeability |
| Ruan et al. | 2022 | Improved gait performance | Evan blue: ⇩ BBB permeability in SN WB: ⇧ ZO-1, claudin-5 and occludin in SN  ⇩ MMP-2/-9  RT-PCR: ⇧ ZO-1, claudin-5 and occludin in SN  ⇩ MMP-2/-9  IHQ: ⇧ TH counts, ZO-1, claudin-5 and occludin in SN and TH density in the CPu  ⇩ MMP activity | Neuroprotective and decreased BBB permeability |
| Bhatia et al. | 2023 | Open field: hyperactive/anxiety-like behaviors in male  Y-maze: female PD PLX mice had greater entries into the novel arm, fewer entries into familiar arms, spent greater time exploring the novel arm, and entered the novel arm earlier | WB: ⇩ monomeric, higher molecular mass, pSer129, and pan α-syn in male   ✖ monomeric, higher molecular mass, pSer129, and pan α-syn in female IHQ: ⇩ pSer129 in the olfactory peduncle and the LLR of male  ✖ pSer129 in the olfactory peduncle and LLR of female  ⇧ average sizes of the pSer129+ inclusions in both sexes | Repopulation is neuroprotective in males |
| Liang et al. | 2023 | NA | IHQ: ⇧TH counts in the SNc  ELISA: ⇩ TNFα and IL-6, ✖ TGFβ FACS: ⇩ microglia (CD11b+CD45lo), infiltrated inflammatory macrophages (CD11b+CD45hi and Ly6CloCD11b+CD45hi), and MHCII+ | Neuroprotective |
| Pereira et al. | 2023 | Pole test: ⇧ time to climb down the pole | IHQ: ⇩ TH counts in the SNc   ⇩ Iba-1+ cells and Iba-1 and CD68 co-labeling in the SNc and CPu  ⇧ Iba-1 cell body area in the SNc and CPu NanoString RNA: ⇩ genes associated with microglial function (CD68, Cd33, Tmem119, Csf1r, P2ry12), apoptosis (Kras, Casp3, Casp8), matrix remodeling (Mmp12, Mmp14), neural connectivity (Mecp2, Lamp1, Adam10, Atcay), inflammation (Tlr4, Il10ra, Ccr2, Il6ra, Tnfrsf1b) and Nox2 (Cybb) | Worsened degeneration and motor impairment |
| Ma et al. | 2024 | NA | IHQ: ⇧ TH counts in the SNc   ⇩ iron deposition in the SNc  ⇩ Iba-1 in the SNc WB: ⇧ glutathione peroxidase 4 (GPX4) | Neuroprotective |
| Stoll et al. | 2024 | NA | IHQ:  *2 mo of treatment*  ✖ number of pSyn+ neurons within the SNc  ✖ TH counts in the SNc  ✖ MHC-II counts in the SNc  ✖ microglial soma size in the SNc  ✖ Cd74, Csf1r, Cxcl10, Fcer1g, Grn, Rt1-a2 and Tyrobp in the SNc *6 mo of treatment*  ⇧ number of pSyn+ neurons within the SNc and ✖ in the CPu  ✖ TH counts in the SNc  ✖ MHC-II counts in the SNc  ⇧ MHC-II expression in the extranigral mesencephalon  ⇧ microglial soma size in the SNc *Pre-treatment*   ✖ number of pSyn+ neurons within the SNc | No effect after short protocol treatment, but proinflammatory microglia phenotype and increased synuclein after long protocol treatment |
| Thi Lai et al. | 2024 | Rotarod: no effect Wire hanging: no effect Clasping test: ⇩ hindlimb clasping score | IHQ: ⇩ pSer129 in different brain regions (CPu, amygdala, and SNc) at 90 d after PFF injections  ✖ pSer129 at 7 and 14 d after PFF injections  ⇧ TH counts in the SNc at 90 d after PFF injections WB: ✖ α-syn soluble and insoluble at 14 d after PFF injections | Neuroprotective |
| Iba et al. | 2025 | Open field: ✖ total activiy, ambulatory moves, and path lenght Wire hang test: ✖ time to fall | RT-PCR: ⇩ Tnf, IL-1β, IL-6, Cxcl2, and Csf-1r  ✖ Ccl2  ⇧ synaptophysin, Snap25, Arc, Arg1, Glb/SAβgal IHQ: ⇩ Iba-1 number in the cortex, HPC, and CPu  ✖ GFAP in the cortex, HPC, and CPu  ✖ CD3, CD4, and CD8 in the cortex, HPC, and CPu  ✖ α-syn density (Syn211) in the cortex, HPC, and CPu  ⇩ α-syn density (pSer129) in cortex and HPC  ⇧ NeuN and synaptophysin (Sy38) in cortex and HPC WB: ⇩ Iba-1 in brain  ⇩ Tx-insol α-syn   ⇩ caspase 3  ⇧ Snap25 ELISA: ✖ α-syn  ⇩ Tx-insol α-syn Morphology: ⇧ size of cell body and cell area in both control and PD groups  microglial RNAseq: ⇩ inflammatory response-associated genes: Tnf, Tnfaip3 and Mpo; proinflammatory cytokines: Tnf, Ccl4, and Cxcl2; and microglial migration, phagocytosis, and proliferation | Neuroprotective |
| Zhang et al. | 2025 | Cylinder test: ⇩ forepaw asymmetry Rotarod: ⇧ time spent on the wheel  Open field: ⇧ average speed | IHQ: ⇧ TH and NeuN counts in the SNc   ⇧ TH density in the CPu  ⇩ pSer129 and p-α-syn+/α-syn+ in the SNc  ⇩ Iba-1 counts in the SNc  ⇩ GFAP and C3  WB: ⇩ GFAP and C3, ⇧ CCN2 and CCN3 RT-PCR: ⇧ CCN2 and CCN3 Midbrain RNAseq: ⇩ pathways related to immune and inflammatory responses, and homeostatic microglial genes  ⇧ extracellular matrix pathway | Neuroprotective |
| *Alzheimer’s disease* | | | | |
| Dagher et al. | 2015 | *6 wk of treatment:* NPRT: ⇧ index NORT: no effect *3 mo of treatment:*  MWM: faster escape latencies, trended towards a faster latency to get to the platform location, and ⇧ platform crosses  NPRT: ⇧ index NORT: no effect | IHQ: larger cell sizes, ⇩ Iba1 in HPC, subiculum, and thalamus  ✖ GFAP/S100+ cells in the HPC  ✖ total human tau accumulation, p-Tau at S202/T205 in the HPC  ELISA: ✖ IL-1β or ApoE   ⇧ CXCL1 and TNFα  ✖ Aβ40 and Aβ42 soluble and insoluble ThioS: ✖ number of plaques, average plaque size, or distribution of small, medium, or large plaques 3D analysis: ⇩ number of microglia associated with a plaque  ✖ caspase 3+ microglia near plaques | Improves cognition, no effect in plaque burden |
| Asai et al | 2015 | NA | *Injected mice:* IHQ: ⇩ AT8+ p-Tau granule cell layer (GCL) of the dentate gyrus ELISA: ✖ hTau in the medial entorhinal cortex RT-PCR: ⇩ Tnfa, Il1b, and Il6 (pro-inflammatory cytokines) and Il10 and Tgfb1 (anti-inflammatory cytokines) in the HPC Electrophysiology: ⇧ population spike amplitude in the dentate granule cell layer *PS19 mice:* IHQ: ⇩ AT8+ p-Tau in the entorhinal cortex and dentate gyrus Exosomes isolation: ⇩ T22+ oligomeric hTau in brain fraction  ⇩ hTau transferention to neurons ex vivo | Reduced tau pathology |
| Spangenberg et al. | 2016 | Contextual fear conditioning: trended toward increased time freezing | *10-mo-old (During and after), 1.5-mo-old (Before) with PLX3397, and 14-mo-old (After) with PLX5622* ThioS and IHQ: ⇩ plaque-associated microglia IHQ: ⇩ Iba-1 counts in cortex, HPC, and thalamus  ✖ 6E10 - diffuse plaque load or size ThioS: ✖ plaque numbers or average plaque size ELISA: ✖Aβ1–38, Aβ1-40, and Aβ1-42 *10-mo-old (During and after)* mRNA: ⇩ ApoE, Cstc, and Cstd (lipid metabolism and protein degradation)  ✖ App, Bace1, or ADAM10 (AD-related signalling)  ⇩ C1qa, Ccl3, Casp3, Csf1r, fcgr4, Ifi204, Itgb2, Olfml3, Tgfb1, Tgfbr1, Tgfbr2, Trem2, Tlr2, Tlr3, Tlr4, Tlr7, Siglech, Pycard, Pdcd1, nirp3, Mrc1 (inflammation-related genes) WB: ✖ APP  IHQ: ⇩ GFAP in cortex  ✖ GFAP in HPC, S100B and Aldh1L1 in either brain regions Golgi: ⇧ total spine density and mushroom spines Cresyl violet: ⇧ neuronal number | No effect on amyloid deposition, neuroprotection |
| Sosna et al. | 2018 | Elevated plus maze: no effect  Y-maze: no effect in the total number of arm entries Contextual fear conditioning: ⇧ time freezing | IHQ: ⇩ amount of intraneuronal amyloid and amyloid plaques  smaller and more compact plaques Dot blot: ⇩ mOC23 and mOC78 (soluble fibrillar amyloid) in the brain  ⇩ mA11–204 (amyloid oligomers) in plasma | Reduced amyloid deposition |
| Unger et al. | 2018 | MWM: no effect | ThioS: ✖ plaques number and size in HPC and cortex Flow cytometry: ⇩ CD11b+/CD45low (microglia) in both sexes  ⇩ CD11b+/CD45high (macrophages) in females IHQ: ⇩ Iba-1 counts in HPC and cortex   ⇩ Iba1+/TMEM119+ (microglia) in the HPC and cortex  ⇩ Iba1+/TMEM119− (macrophages) in the HPC and cortex  ⇩ Iba1+/ThioS+ cell numbers (phagocytosis) in the HPC and cortex percentage  ✖ % of Iba1+/ThioS+ cells in the total Iba1+ cell counts   ✖ Iba1+/TMEM119+/ThioS+ colocalization   ⇩ Iba1+/TMEM119+/CD68 total number in both regions  ⇩ Iba1+/TMEM119-/CD68 total number in both regions  ⇧ in the numbers of CD3+, more specifically CD3+/CD8+ T-cells  CD8+ T-cells interacts with Iba1+/TMEM119+ cells RT-qPCR: ⇩ AIF1, TMEM119, H2-Aa, IL6, MRC1, TGFbeta, Trem2, and CD33 in the HPC and cortex and IL-10 in HPC | No effect on amyloid deposition |
| Spangenberg et al. | 2019 | Elevated plus maze: ⇧ time in open arms and ⇩ time in closed ones Open field: no effect Spontaneous Alternation Y-Maze: no effect MWM: no effect Contextual fear conditioning: no effect | *PLX5622 - 10 wk treatment:* ThioS: ⇩ plaque number in cortex and thalamus ELISA: ✖ Aβ38, Aβ40, or Aβ42, in detergent-soluble or -insoluble cortical and thalamic *24 wk treatment:* ThioS: ⇩ plaque number and volume in cortex and thalamus, plaque number in subiculum   ⇩ plaque circularity and intensity in cortex, thalamus, and subiculum IHQ: ⇩ GFAP in cortex  ⇧ cerebral amyloid angiopathy- plaques positive for protofibrils (OC) and Aβ1-42, negative for oligomers (A11 antibody)  ⇩ pyroglutamate-3 Aβ (plaque core) and 6E10+diffuse plaques  ⇩ Claudin-5 staining in Aβ-associated blood vessels  ⇩ coaggregation of ApoE with Aβ fibrils and colocalization of ApoE and Iba-1  ⇧ LAMP1 and APP volume inside plaques in subiculum (dystrophic neurites) ELISA: ✖ Aβ38, Aβ40, or Aβ42, in detergent-soluble or -insoluble cortical and thalamic WB: ⇧ full length (fl)   ✖ APP and Carboxy-terminal fragments of APP (C99 and C83) *PLX3397* ThioS: ✖ plaque number  ⇧ cerebral amyloid angiopathy *PLX5622- 24 wk treatment* RNAseq: ⇩ Csf1r, Cx3cr1, C1qa, Hexb, Siglech, and Spi1 in cortex, HPC, and thalamus+CPu  ⇩ Ccl6, Clec7a, Cst7, Ctsd, Ctsz, and Itgax Asb10, B2m, Ccl3, Ch25h, Gpr65, Grn, Hcar2, Hexa, Ly9, Lyz2, Oasl2, Pdcd1, Plcg2, and Treml2 (markers of disease-associated microglia - DAM)  In HPC, the levels were higher than the other brain regions, suggesting the surviving microglia observed in the subiculum are plaque-forming/associated microglia  ⇧ synaptic and neuronal genes (such as Dlk2, Dync1l1, Gls, Kcnq3, Nrg3, and Scn1b)  ✖ genes associated with APP processing, Aβ clearance and metabolism *Repopulation*  plaque numbers were equal to the untreated 5xFAD mice  ⇩ average plaque volumes   GFAP+ associated with plaques and vascular deposition of Aβ are present | Reduced amyloid deposition and plaque formation and downregulated neuronal genes Repopulation: restoration of plaque pathology |
| Shi | 2019 | NA | *TE4 mice (ApoE present)* Sudan black staining: ⇧ HPC, piriform/entorhinal cortex, and amygdala volume IHQ: ⇩ CD68 staining in the HPC and entorhinal/piriform cortex  Shift to type1 early p-Tau (AT8+) staining pattern - earliest pathological tau stage - in the HPC  ⇩ p-Tau area in the HPC  ⇧ intensity of apoE signal that largely co-localized with GFAP+ astrocytes  ⇧ diffuse apoE staining  ⇧ apoE+ neuronal shaped, not co-localize with NeuN in dentate gyrus, CA3 of the HPC, and the piriform/entorhinal cortex, but partially co-localized with p-Tau-positive neurons in dentate gyrus ELISA: ⇩ p-Tau levels and ⇧ insoluble tau level in posterior cortex  ⇧ soluble apoE levels WB: ⇧ soluble apoE levels *TEKO mice (ApoE absence)* IHQ: ⇩ CD68 staining in the HPC and entorhinal/piriform cortex  Type1 p-Tau still present, but shift to type1-Early - solely mossy fiber staining - in the HPC  ⇧ intensity of apoE signal that largely co-localized with GFAP+ astrocytes  ⇧ diffuse apoE staining ELISA: ⇩ p-Tau levels in posterior cortex  ⇧ soluble apoE levels WB: ⇧ soluble apoE levels | Prevented brain atrophy and reduced tau pathology |
| Casali et al. | 2020 | NA | Depletion:  IHQ: ⇩ 6E10 area in the thalamus and cortex  ✖ 6E10 area in the HPC and subiculum  ⇩ Iba-1 counts and area  ⇧ n-terminal APP and ubiquitin coverage in dystrophic neurites in the cortex and HPC ThioS + IHQ: ⇩ 6E10+/ThioS+ (‘compact plaques’) in cortex, HPC and subiculum; ⇧ 6E10+ (‘diffuse/filamentous’ plaques) in the subiculum; ✖ ThioS+(dense-core plaques) ELISA: ✖ Aβ soluble and insoluble  RT-qPCR: ⇩ Iba1, Trem2, Csf1r, Tnfa, Il6, and Il1b  ⇩ Spp1, Tgfbr1, Mafb, Pu.1, Axl, and Mertk (microglial homeostasis) *Repopulation:* IHQ: ✖ Iba-1 counts and area HPC, subiculum, and thalamus; ⇩ in the cortex  ✖ 6E10 area in all regions  ✖ n-terminal APP and ubiquitin coverage in dystrophic neurites in the cortex and HPC ThioS + IHQ: ✖ 6E10+/ThioS+ (‘compact plaques’) in HPC, subiculum and thalamus but ⇩ 6E10+/ThioS+ in the cortex; ✖ 6E10+ (‘diffuse/filamentous’ plaques) in the subiculum; ⇩ThioS+(dense-core plaques) in thalamus RT-qPCR: ✖ Iba1, Trem2, Csf1r, Tnfa, Il6, and Il1b  ✖ Spp1, Mafb, Pu.1 (microglial homeostasis)  ⇩ Tgfbr1, Axl, and Mertk (microglial homeostasis) | Alters plaque morphologies (from compact to diffuse-like plaque) and enhances neuritic dystrophy |
| Crapser et al. | 2020 | NA | *5xFAD:* IHQ: ⇧ perineuronal net area in subiculum and visual cortex ThioS: ⇩ plaques in the visual cortex *3xTg:* IHQ: ⇧ perineuronal net area in subiculum and visual cortex ThioS: ✖ plaques in the visual cortex | Preserve perineuronal nets |
| Michael et al. | 2020 | NA | IHQ: ⇩ Iba-1 and TMEM119 in HPC and cortex  ⇩ FLAP, 5-Lox+/Iba1+ in HPC and cortex RNAseq: ⇩ Mrc1, TMEM119, CD33 and Aif1 (microglial genes)  ⇩ Alox5ap, Cysltr1, and Alox5 (leukotriene signaling) RT-qPCR: ⇩ Alox5, Alox5ap, and Cysltr1 | Reduces the leukotriene pathway |
| Son et al. | 2020 | NA | WB: ⇩ full-length amyloid precursor protein, carboxyl-terminal fragment and Aβ in the cortex  ⇩ Aβ in the HPC  ⇩ Iba-1 in the HPC and cortex  ⇧ synaptophysin and PSD-95 in the HPC and cortex Cresyl violet: prevented CA1 decrease in thickness  PET imaging: ⇧ dopamine D2 receptor (D2R) and ✖ metabotropic glutamate receptor 5 (mGluR5) IHQ: ⇧ D2R and TH | Reduced amyloid deposition |
| Benitez et al. | 2021 | NA | IHQ: ⇩ Iba-1 CD68+ in the HPC  ⇧ proportion of the remaining microglia that were CD68+ in the HPC  ✖ percentage plaque coverage and density of plaques in the HPC  ⇩ density only of small plaques in treated AppNL-G-F mice, but not large plaques Patch-clamp: ⇩ paired-pulse ratio (exacerbates the App knock-in phenotype) | No effect overall in plaque load, worsened synaptic function |
| Bennett et al. | 2021 | NA | RT-qPCR: ⇧ Cd68 and Tgf1β  ✖ ApoE, CX3CR1, IL1B, TREM2  ⇧ Plau and ✖ Serpine1 (blood vessel morphology) Cell-based tau seeding activity assay: ✖ seeding activity of tau ELISA: ✖ total tau WB: ✖ pP38/total P38, pS202/T205, and pT231 (Tau isoforms) IHQ: ✖ number of tau+ neurons (pS202, T205) in the cortex  ✖ NeuN counts in the cortex  ✖ vascular density in the cortex  ✖ GFAP | No effect in tau pathology nor in degeneration |
| Delizannis et al. | 2021 | NA | IHQ: ⇩ Iba1+ and Iba1+ cells associated with Aβ plaques  ⇩ Aβ plaque density (NAB228-positive) in the cortex  ✖ Aβ plaque density (NAB228-positive) in the subiculum  ⇩ integrated Aβ plaque signal and area per plaque on cortical layers 4–6  ⇩ APP-positive dystrophic processes (neuritic dystrophy)  ⇩ tau (AT8-positive) in the cortex  ✖ tau (AT8-positive) in the subiculum  ⇩ integrated APP value per plaque in the cortex | Reduced amyloid deposition and plaque burden; attenuated tau pathology |
| Tsai et al. | 2021 | NA | *PLX treatment and withdraw:* RT-qPCR: ⇩ inositol polyphosphate-5-phosphatase (regulation of microglial gene expression) in the cortex | NA |
| Dodiya et al. | 2021 | NA | IHQ: ⇩ Aβ plaque burden after 9 wk of treatment  ✖ Aβ plaque burden after 3 mo of treatment | Reduced plaque burden only in the short treatment. |
| Clayton et al. | 2021 | NA | ThioS: ⇩ circularity of dense-core plaques in the cortex  ⇧ Aβ plaque area, number, and size in the cortex  ⇩ sphericity and ⇧ of plaque volume and area in the cortex IHQ: ⇩ P2RY12+ area  4G8 (Aβ17-24): ⇧ size of plaques and overall plaque area in the cortex  82E1 (Aβ1–16) : ✖ size, circularity, number and overall plaque area in the cortex  ✖ GFAP  ⇩ p-Tau (pSer202/pSer205)-positive cells from granular cell layer/ p-tau+ from medial entorhinal cortex (tau propagation)  ⇧ %AT8+ plaque area  ⇩ Clec7A+ microglia  ⇩ double staining-Clec7A+Tsg101+ (marker of extracellular vesicles) | Reduced propagation of p-tau, but increased plaque burden |
| Lodder et al. | 2021 | NA | scRNA-Seq on CD45 + immune cells from brains: remaining microglia exhibited a DAM or reactive phenotype and ⇧ Apoe  IHQ: ✖ Aβ area (W02) in cortex and HPC  ⇩ p-tau area (AT8) in cortex and HPC  ⇧ cortical area and hippocampal volume | No effect in plaque burden, but reduces tau pathology and neurodegeneration |
| Karaahmet et al. | 2022 | Open field: no effect NORT: no effect Lashley maze: no effect Contextual fear conditioning: no effect | Repopulation: IHQ: ✖ area fraction occupied by amyloid plaques, the average size and number of plaques in the subiculum  ✖ dense-core plaques (anti-MeX04) in the subiculum  ✖ neuritic damage (anti-LAMP1) in all, but female APP/PS1 that showed ⇧ LAMP1 in the subiculum  ✖ %Iba1 at plaque, total microglial coverage   ✖ %CD68+ microglial volume   ✖ P2RY12 density in APP/PS1  ⇩ P2RY12 density in 3xTg  ⇧ TMEM119 density in female APP/PS1   ⇧ pT205, ⇩ pS409 ELISA: ✖ Aβ42 and Aβ40 soluble and Aβ40 insoluble forms  Flow cytometry/FACS: ✖ %MeX04+microglia (plaque-phagocytosing microglia) single-cell RNAseq: identification of a PLX-microglia cluster with ⇧ Cxcl13 ELISA: ⇧ CXCL13 RNAScope: ⇧ Cxcl13 mRNA | Repopulation: no effect on plaque pathology, reduced tau pathology |
| Wendt et al. | 2022 | NA | IHQ: ✖ plaque load/number in cortex   ⇧ MAP2+ neurons in cortex   ⇧ redox ratios in dystrophic neurites (oxidative stress)   ⇧ LAMP1 in dystrophic neurites  ⇩ Iba-1 area/LAMP1 area   ⇧ LAMP1 ratio (dystrophic neurites to surrounding neurons) | No effect on plaque load, but neuroprotective |
| Gaunt et al. | 2023 | NA | ELISA: ⇧ CSF1 in both regions  ⇧ IL6 and IL27, ⇩ BAFF and CCL3 in the DCN  ✖ IL6 and IL27, ⇧ IL33, CCL3 and CCL4 in the EC  ✖ TNF in both regions IHQ: ✖ fibrillar Aβ (FSB-positive) plaque counts, total area or average size in EC  ⇩ FSB-positive plaques counts and area in the DCN   ✖ 6E10 staining in both regions | Depleted fibrillar plaques in the DCN, no effect in EC; increased inflammatory cytokines |
| Son et al. | 2023 | NA | RT-PCR: ⇩ Csf1r, CD68, Ccl6, IL-1β, IL-6, and IL-4 in the HPC  ⇩ Trem2, Fcgr1, Ctss, and Spi1 in the HPC | Reduced inflammation |
| Weigel et al. | 2023 | Jet lag re-entrainment trials: no effect | NA | NA |
| Johnson et al. | 2023 | Automated home-cage monitoring: ⇩ in hyperactivity, normalized the amounts of wheel running and active time | Tau-prion bioassay: ⇩ tau-prion activity in the forebrains in all treatments time and type  ✖ tau-prion activity in the hindbrain in the acute and interventional treatment  ⇩ tau-prion activity in the hindbrain in the chronic and terminal treatment  ⇩ tau-prion activity in the ipsilateral forebrain and hindbrain, as well as in the contralateral forebrain (mice with inoculated fibrils) ELISA: ⇩ pS396 tau in the forebrain and hindbrain after PLX3397 acute, chronic, or terminal treatment  ⇩ pT231 tau in the interventional and intermittent treatment in the forebrain IHQ:⇩ pS202/T205 tau in forebrain for acute and chronic treatment   ⇧ NeuN in forebrain in acute treatment, and ✖ NeuN in neither acute, chronic, and terminal in the hindbrain and chronic and terminal in the forebrain Survival rate: ⇧ the median survival of female, and ⇩ weight loss Plasma immunoassay: ⇩ plasma levels of neurofilament light chain in females, but ⇧ in males in the terminal treatment and in mice with inoculated fibrils  ✖ plasma levels of neurofilament light chain in females, but ⇩ in males in the intermittent treatment Transcriptomic analysis: PLX-treated Tg2541 mice showed a greater correlation with wild type mice Hydrophilic interaction liquid chromatography tandem mass spectrometry (HILIC-MS/MS)-based metabolomics: ⇧ brain levels of glutamate and GABA only in male mice; ✖ acetylcholine, glycine, or histamine RT-PCR: ⇩ A1 astrocytic gene Bioluminescence imaging with GFAP-luciferase: ⇩ signal in both the forebrain and the hindbrain Microglia morphology: ⇧ number of branches per cell, total process length, and territory size  Microglia transcriptomic analysis: ⇩ genes expression increased on AD group; ⇧ activation of inflammation-related pathways in PLX-treated male mice compared to female mice (tau-activated NFkB and excitotoxic pathways, but not amyloid induced inflammasome genes) | Reduced pathogenic tau and neuroprotective after acute treatment |
| Wang et al. | 2023 | *Depletion:*  MWM: no effect Fear-conditioning test: no effect T-Maze test: no effect  *Repopulation (1 mo):* MWM: ⇩ time to locate the platform  ⇧ times crossed the target platform  ⇩ time searching for the platform Fear-conditioning test: ⇧ freezing time during contextual testing  ✖ freezing time during cued testing T-Maze test: trend towards increased alternations | *One month repopulation:* WB: ⇧ NMDA receptor subunits (NR2A and NR2B), AMPA receptor subunit (GluR1), PSD95, synaptophysin in the hippocampi  ⇧ BDNF, p-TrkB IHQ: ⇧ number of puncta for PSD95 and vGluT1  ⇧ density of vGluT1-PSD95 colocalization (synaptic contacts) in the HPC, ✖ in the cortex  ✖ total area or plaque load in the HPC  ⇩ microglia engulfment of PSD95 (Iba1, CD68, PSD95 staining)  ⇧ DCX-positive neurons in the dentate gyrus  ⇧ BNDF area within microglia cells, but not within astrocytes Microglia morphology: restored number of intersections, endpoints, and processes in HPC, less effect in cortex Golgi staining: ⇧ density of mature spines (mushroom morphology) Patch clamp: restored long-term potentiation (LTP) ELISA: ✖ Aβ40 and Aβ42 soluble and insoluble RT-PCR: ⇩ iNos, Ccl3, Ccl4, Ccl6, and B2m; ✖ Trem2, Apoe, Cd11c, and Cd9 in the HPC  ✖ IL6, TNF, IL1β, NGF, neurotrophin 3 and 4 in the HPC  ⇧ BDNF in the HPC Microglia RNAseq: expression profile closely resembling that of microglia from WT mice, enrichment within the neurotrophic signaling pathway  *Three month repopulation:* IHQ: ⇩ number and deposition of Aβ plaques  ⇩ Iba1 *Eight month repopulation:* IHQ: ✖ deposition of Aβ plaques  ✖ Iba1, PSD95 and Synapsin *Depletion:* WB: ✖ NR2A, NR2B, GluR1, GluR2, PSD95, synaptophysin in the HPC | Repopulation rescues cognitive, synaptic impairments, and increased neurotrophic factors, but no effect in plaque burden; no effect of depletion |
| Kodali et al. | 2025 | NA | IHQ: ⇩ microglial number around plaques (Aβ-42 and Iba1 co-staining) in HPC and cortex  ⇩ Clec7a expression in plaque-associated microglia in the HPC   ⇩ %CD68+ microglia in the cortex and HPC  Morphology analysis: ⇧ total process length and but ✖ total number of nodes and endings in the HPC, ⇧ total process length and number of nodes and endings in the cortex, ⇧ number of intersections, ⇧ length of the processes, and ⇧ number of nodes and endings at multiple distances from the soma in the HPC and cortex  ⇩ area fraction of NLRP3 and apoptosis-associated speck-like protein containing a CARD (ASC) complexes in the microglia in both HPC and cortex   ⇩ area fraction of GFAP in CA3 of HPC, but ✖ GFAP in DG, entire HPC, and cortex  ⇩ area fraction of pS6 within NeuN+ and Iba1+ in cortex and within Iba+ in HPC, but ✖ pS6/NeuN in HPC   ⇩ % neurons with pS6 cortex, but ✖ in HPC  ✖ % microglia with pS6 cortex and in HPC  ⇩ area fraction of p62 within microglia in hippocampal and cortex   ⇩ area fraction of p62 within neurons in cortex   ✖ area fraction of p62 within neurons in hippocampal   ✖ density of Aβ plaques in HPC and cortex  ✖ DCX+ neurons in HPC  ELISA: ⇩ NF-kB-p65, and NLRP3 in the HPC and ⇩ NLRP3, ASC, and cleaved caspase-1 in the cortex   ⇩ IL-1β, IL-18, MIP1α in the HPC and in the cortex  ⇩ phospho-and//pan-mTOR levels in the HPC and cortex  ⇧ beclin-1 and ATG-5 in HPC, but ✖ in cortex  ✖ soluble Aβ-42 in HPC and cortex | No effect in plaque burden, but reduce inflammation and autophagy |

Unless indicated, comparations where made between AD or PD-vehicle with AD or PD-PLX treated groups. *Data was estimated from the graphs. Abbreviations: 4G8: anti-amyloid-β antibody that recognizes residues 18-23; 5-HIAA: 5-hydroxy indoleacetic acid; 5-HT: 5-hydroxytryptamine or serotonin; 5-Lox: 5-lipoxygenase; 6E10: anti-amyloid-β antibody that recognizes N-terminal region, specifically residues 1-16; 82E1: anti-human amyloid-β antibody that recognizes N-terminal region; A11: anti-amyloid-β antibody that recognizes oligomer A11; AD: Alzheimer’s disease; Adam10/ADAM10: A Disintegrin and Metalloproteinase 10; AIF1/Aif1: Allograft inflammatory factor 1; Aldh1L1: Aldehyde Dehydrogenase 1 Family Member L1; Alox5: Arachidonate 5-Lipoxygenase; Alox5ap: Arachidonate 5-Lipoxygenase Activating Protein; Tnfaip3: Tumor necrosis factor alpha-induced protein 3; AMPA: α-amino-3-hydroxy-5-methyl-4-isoxazolepropionic acid; ApoE: Apolipoprotein E; App/APP: Amyloid-beta precursor protein; Arc: Activity-regulated cytoskeleton-associated protein; Arg1: arginase; Asb10: Ankyrin Repeat And SOCS Box Containing 10; AT8: anti-tau protein antibody that recognizes phosphorylated tau at both serine 202 and threonine 205; Atcay: Cayman cerebellar ataxia gene; ATG-5: Autophagy-Related Gene 5; Axl: Anexelekto, tyrosine kinase receptor; Aβ1–16/ Aβ17-24/ Aβ38/ Aβ40/ Aβ42: amyloid beta 1-16, 17-24, 38, 40, or 42 peptide; B2m: beta2-Microglobulin; Bace1: Beta-site Amyloid Precursor Protein Cleaving Enzyme 1; BAFF: B-cell activating factor; Bax: Bcl-2-associated X protein; BBB: blood-brain barrier; Bcl-xL: B-cell lymphoma-extra-large; BDNF: Brain-derived neurotrophic factor; C1q: Complement component 1q; C1qa: complement component 1, q subcomponent, A chain; C3: complement component 3; C83: Diffuse large B-cell lymphoma; C99: Other malignant neoplasms of lymphoid, hematopoietic and related tissue; Casp3: caspase 3; Casp8: caspase 8; CCL2 / Ccl2: Chemokine (C-C motif) ligand 2 (also known as MCP-1, monocyte chemoattractant protein-1); CCL22: Chemokine (C-C motif) ligand 22; Cxcl2: Chemokine (C-X-C motif) ligand 2; CCL3/ Ccl3: Chemokine (C-C motif) ligand 3; CCL4/ Ccl4: Chemokine (C-C motif) ligand 4; Ccl6: Chemokine (C-C motif) ligand 6; Ccn2: Cellular communication network factor 2; Ccr2: C-C chemokine receptor type 2; Cd11c: Integrin alpha X; CD33/ Cd33: Sialic acid-binding Ig-like lectin 3; CD45: Protein tyrosine phosphatase receptor type C; CD68: Cluster of differentiation 68; Cd74: Cluster of Differentiation 74; Cd9: Cluster of differentiation 9; Ch25h: Cholesterol 25-hydroxylase; Clec7a: C-type lectin domain family 7 member A; COX2: Cyclooxygenase-2; CPu: striatum; CSF1: Colony Stimulating Factor-1; Csf1r: Colony Stimulating Factor-1 receptor; Cst7: Cystatin F; Cstc: Cystatin C; Cstd: Cystatin D; Ctsd: Cathepsin D; Ctss: Cathepsin S; Ctsz: Cathepsin Z; Cx3cr1/ CX3CR1: CX3C chemokine receptor 1; Cxcl10: Chemokine (C-X-C motif) ligand 10; CXCL13: Chemokine (C-X-C motif) ligand 13; MAP2: Microtubule-associated protein 2; Cybb: Cytochrome b-245 beta chain; Cysltr1: Cysteinyl leukotriene receptor 1; d: day or days; DA: dopamine; DCN: deep cerebellar nuclei; DCX: doublecortin; Dlk2: Delta-like 2 homolog; DOPAC: 3,4-Dihydroxyphenylacetic acid; Dync1l1: Dynein cytoplasmic 1 light intermediate chain 1; EC: Entorhinal cortex; ELISA: Enzyme-Linked Immunosorbent Assay; FACS: Fluorescence-Activated Cell Sorting; Fcer1g: Fc epsilon receptor Ig; Fcgr1: Fc gamma receptor I; Fcgr4: Fc gamma receptor IV; FLAP: 5-Lipoxygenase Activating Protein; GABA: Gamma-Aminobutyric Acid; GFAP: Glial Fibrillary Acidic Protein; Glb / SAβgal: β-galactosidase; Gls: Glutaminase; GluR1: Glutamate Receptor 1; GluR2: Glutamate Receptor 2; Gpr65: G protein-coupled receptor 65; Grn: Granulin; GSH: Glutathione; H2-Aa: Histocompatibility 2, class II antigen A, alpha; Hcar2: Hydroxycarboxylic acid receptor 2; Hexa: Hexosaminidase A; Hexb: Hexosaminidase B; HPC: Hippocampus; hTau: Human Tau protein; HVA: Homovanillic Acid; Iba-1: Ionized calcium-binding adapter molecule 1; Ifi204: Interferon-activable protein 204; IFNγ: Interferon gamma; IGF1: Insulin-like Growth Factor 1; IHQ: Immunohistochemistry; IL: Interleukin; iNOS: Inducible Nitric Oxide Synthase; IP-10: Interferon gamma-induced protein 10; Itgax: Integrin alpha X; Itgb2: Integrin beta 2; Kcnq3: Potassium voltage-gated channel subfamily Q member 3; Kras: Kirsten rat sarcoma viral oncogene; Lamp1 / LAMP1: Lysosomal-associated membrane protein 1; LC: Locus Coeruleus; LLR: Lateral Limbic Rhinencephalon; Ly9: Lymphocyte antigen 9; Lyz2: Lysozyme 2; Mafb: MAF bZIP transcription factor B; MDA: Malondialdehyde; Mecp2: Methyl-CpG binding protein 2; Mertk: MER proto-oncogene tyrosine kinase; MeX04: Methoxy-X04 (Amyloid-binding dye); MHC I: Major Histocompatibility Complex Class I; MHC II: Major Histocompatibility Complex Class II; MMP/ Mmp12 / Mmp14 / MMP-2/-9: Matrix Metalloproteinases (12, 14, 2, 9); mo: month or months; mOC23/ mOC78: Anti- soluble fibrillar amyloid; MRC1/ Mrc1: Mannose Receptor C-Type 1; mTOR: Mammalian Target of Rapamycin; MWM: Morris Water Maze; MPO: Myeloperoxidase; NA: Not available; NAB228: anti-amyloid peptides antibody; NeuN: Neuronal Nuclei marker; NFkB: Nuclear Factor kappa-light-chain-enhancer of activated B cells; NGF: Nerve Growth Factor; NLRP3: NOD-, LRR- and pyrin domain-containing protein 3; NMDA: N-Methyl-D-aspartate; NORT: Novel Object Recognition Test; NPRT: Novel Place Recognition Test; Nox2: NADPH oxidase 2; NR: Not reported; NR2A/NR2B: NMDA Receptor Subunit 2A / 2B; Nrg3: Neuregulin 3; Oasl2: 2'-5'-Oligoadenylate Synthetase-Like 2; Olfml3: Olfactomedin-like 3; P2ry12/P2RY12: Purinergic Receptor P2Y12; P38: Mitogen-activated protein kinase; PD: Parkinson's Disease; Pdcd1: Programmed Cell Death Protein 1; PET: Positron Emission Tomography; Plcg2: Phospholipase C gamma 2; pS2: Estrogen-regulated protein; PSD95: Postsynaptic density protein 95; p-Tau: Phosphorylated Tau protein; p-TrkB: Phosphorylated Tropomyosin receptor kinase B; Pycard: PYD and CARD domain-containing protein; RNAseq: RNA Sequencing; Rt1-a2: Rat MHC class I gene; RT-PCR: Reverse Transcription Polymerase Chain Reaction; S100/S100B: S100 calcium-binding protein B; Scn1b: Sodium channel, voltage-gated, type I, beta subunit; Siglec / Siglech: Sialic acid-binding Ig-like lectins; SN: Substantia Nigra; Snap25: Synaptosomal-associated protein; SNc: Substantia Nigra pars compacta; Spp1: Secreted phosphoprotein 1; Tgf1β/TGF-b/Tgfb1/TGFbeta/TGFβ1: Transforming Growth Factor Beta 1; Tgfbr1/Tgfbr2: TGF-β receptor 1/2; TH: Tyrosine Hydroxylase; ThioS: Thioflavin S; Tlr2/Tlr3/Tlr4/Tlr7: Toll-like receptors 2, 3, 4, 7; Tmem119/TMEM119: Transmembrane protein 119; Tnfa/TNFα: Tumor Necrosis Factor Alpha; Tnfrsf1b: Tumor Necrosis Factor Receptor Superfamily Member 1B; TREM2/Trem2: Triggering Receptor Expressed on Myeloid Cells 2; Treml2: Triggering Receptor Expressed on Myeloid Cells Like 2; Tsg101: Tumor Susceptibility Gene 101; TSPO: Translocator Protein, mitochondrial marker; TUNEL: Terminal deoxynucleotidyl transferase dUTP nick end labeling; Tyrobp: TYRO protein tyrosine kinase binding protein; vGluT1: Vesicular Glutamate Transporter 1; W02: anti-amyloid beta antibody; WB: Western Blotting; wk: week or weeks; ZO-1: Zonula Occludens-1; α-syn: α-synuclein.
